# Concerted Proton-Coupled Electron Transfer to a Graphite Adsorbed Metalloporphyrin Occurs by Band to Bond Electron Redistribution

**DOI:** 10.1021/acscentsci.3c00186

**Published:** 2023-04-19

**Authors:** Phillips Hutchison, Corey J. Kaminsky, Yogesh Surendranath, Sharon Hammes-Schiffer

**Affiliations:** † Department of Chemistry, Yale University, New Haven, Connecticut 06520, United States; ‡ Department of Chemistry, Massachusetts Institute of Technology, Cambridge, Massachusetts 02139, United States

## Abstract

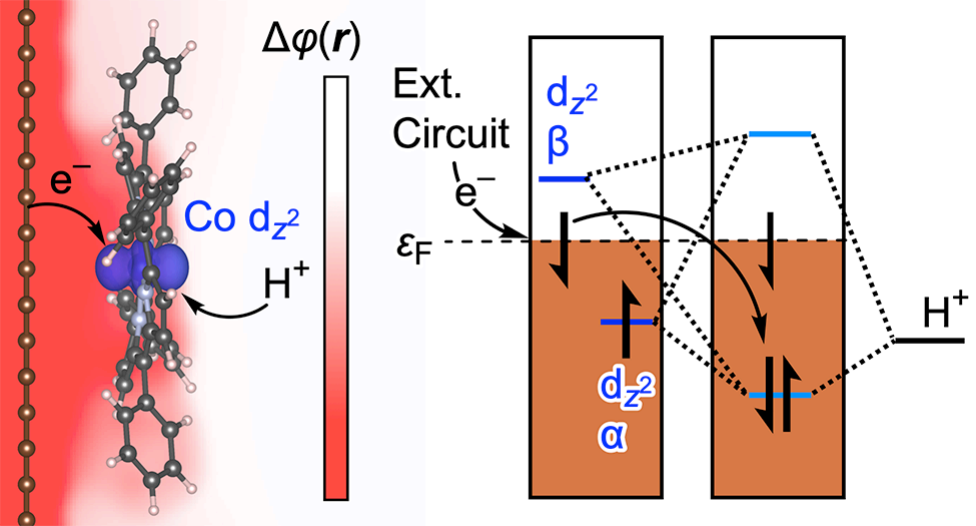

Surface immobilized catalysts are highly promising candidates for a range of energy conversion reactions, and atomistic mechanistic understanding is essential for their rational design. Cobalt tetraphenylporphyrin (CoTPP) nonspecifically adsorbed on a graphitic surface has been shown to undergo concerted proton-coupled electron transfer (PCET) in aqueous solution. Herein, density functional theory calculations on both cluster and periodic models representing π-stacked interactions or axial ligation to a surface oxygenate are performed. As the electrode surface is charged due to applied potential, the adsorbed molecule experiences the electrical polarization of the interface and nearly the same electrostatic potential as the electrode, regardless of the adsorption mode. PCET occurs by electron abstraction from the surface to the CoTPP concerted with protonation to form a cobalt hydride, thereby circumventing Co(II/I) redox. Specifically, the Co(II) d-state localized orbital interacts with a proton from solution and an electron from the delocalized graphitic band states to produce a Co(III)–H bonding orbital below the Fermi level, corresponding to redistribution of electrons from the band states to the bonding states. These insights have broad implications for electrocatalysis by chemically modified electrodes and surface immobilized catalysts.

## Abstract 2

Computations reveal concerted PCET for a cobalt porphyrin adsorbed on a graphitic electrode, where electron abstraction from graphite accompanies protonation of cobalt due to electrostatic coupling.

## Introduction

The interconversion between electrical and chemical energy is a critical element of a renewable energy economy. This interconversion requires catalysts that are scalable and highly tunable, as well as composed of low cost, earth-abundant materials. Molecules bound to electrode surfaces constitute a potent class of catalysts for a variety of key energy conversion reactions.^1−17^ This class of catalysts^14,15,18,19^ displays reactivity that depends on the nature of the surface, the mode of attachment, and the solvent.^13,14,20−23^ Atomistic mechanistic insight into the function of these modified electrodes is key to their rational design.

The catalytic activity of chemically modified electrodes raises numerous fundamental mechanistic and electronic structure questions that lie at the intersection of heterogeneous and molecular catalysis. Within the realm of homogeneous catalysis by soluble molecules, electrical polarization of the electrode drives outer-sphere electron transfer from the electrode to the frontier orbitals of the molecular complex, initiating substrate activation. In contrast, for heterogeneous catalysis by the active sites on bulk metallic surfaces, electrode polarization drives charged solvated species toward the interface, and band electrons localize to form chemical bonds to the adsorbed intermediates. The corresponding picture for molecularly modified electrodes remains unclear, as they consist of both localized orbitals of the immobilized molecule and delocalized bands within the electrode support. Moreover, interactions between the molecule and the electrode surface have been shown to play a significant role in modulating the reaction chemistry of the active site.^20,21^ The mechanism by which interactions between localized molecular orbitals and delocalized bands catalyze bond formation and activation is not yet well understood.

Substantial insight into chemistry at molecularly modified electrodes has been gained by investigating graphite-conjugated catalysts (GCCs), which consist of molecules connected to graphitic electrodes through conjugated aromatic linkages.^24−28^ In GCCs, the strong electronic coupling between the molecule and the electrode allows the molecule to behave as an active site of a metallic surface. Specifically, electrode polarization in these systems alters the occupation of graphitic band states associated with the electrode surface, whereas bond formation and activation at the GCC active site is concerted with electron flow from the electrode surface that populates quasi-molecular electronic states. Moreover, bond formation at the GCC active site does not necessarily alter the local valency of the metal center to which the bond is formed.^29^ Consequently, proton-coupled electron transfer (PCET) at GCC active sites is often decoupled from the redox potential of the molecular analogue.^27^

Recently, the Surendranath group found that a cobalt tetraphenylporphyrin (CoTPP) molecule nonspecifically adsorbed to a graphitic surface can exhibit similar decoupling of its PCET chemistry from the redox potential of the CoTPP.^22^ In aqueous solution, the hydrogen evolution reaction (HER) catalyzed by this system has been shown to proceed through a concerted PCET mechanism with activity that is decoupled from the Co(II/I) redox potential. In contrast, in acetonitrile HER catalysis proceeds through a stepwise redox-mediated mechanism, in which electron transfer (ET) forming Co(I) precedes proton transfer (PT). This transition from a stepwise to concerted PCET mechanism is attributed to the insolubility of CoTPP in water, leading to a stronger interaction between the molecule and the graphitic surface in aqueous media. Thus, the CoTPP system provides an ideal platform for examining the interplay between frontier metal orbitals and graphitic bands in fostering a concerted PCET mechanism for HER catalysis.

Herein, we use computational methods to examine how the interactions between CoTPP and a graphitic surface impact the PCET chemistry of the adsorbed CoTPP. We perform density functional theory (DFT) calculations using both cluster-based and periodic models of CoTPP interacting with graphitic carbon to span a range of support effects. The mode of adsorption is controlled by allowing for either physisorbed π–π interactions or chemisorbed direct axial ligation to a surface oxidic moiety. By controlling both the size of the surface and the mode of interaction, these calculations identify the electronic structure properties that lead to the experimentally observed alteration of the CoTPP PCET mechanism in water compared to acetonitrile. Specifically, we find that, irrespective of the mode of interaction, the adsorbed CoTPP experiences the electrical polarization of the interface, which drives protonation to form a cobalt hydride concerted with electron redistribution from the surface to CoTPP. This concerted band to bond electron redistribution circumvents the Co(II/I) redox process. The fundamental physical insights obtained from these calculations are not specific to CoTPP. The proposed origins for altered reaction chemistry have broad implications for electrocatalysis by chemically modified electrodes in aqueous media, wherein the low solubility of many catalysts forces direct interaction with the electrode surface.

## Computational Methods

Models of metalloporphyrins on graphitic surfaces are computationally challenging even when the surface is modeled as a single defect-free layer of graphene. Periodic DFT models are highly useful to describe the extended nature of the graphitic surface, but these require large unit cells to isolate the adsorbing molecule from lateral interactions with its periodic images and dense k-point meshes to accurately capture the electronic structure of the graphene.^30−32^ Due to the large size of the graphitic layers, multilayer models are computationally expensive. Additionally, Hubbard *U* corrections^33^ must often be used to recover d-orbital separation of the transition metal. Splitting of the d-orbitals is not captured well in commonly used generalized gradient approximation (GGA) functionals that accurately treat the band structure of graphene.^34−36^ Cluster-based models, wherein the graphitic surface is replaced by a large graphitic flake, can greatly reduce the computational cost and allow the use of functionals that accurately treat the transition metal.^37−40^ At computationally efficient cluster sizes, however, the graphitic states are still discrete electronic states rather than a continuum. Our strategy is to use both periodic and cluster-based approaches to obtain a comprehensive picture.^41^

**Figure 1:**
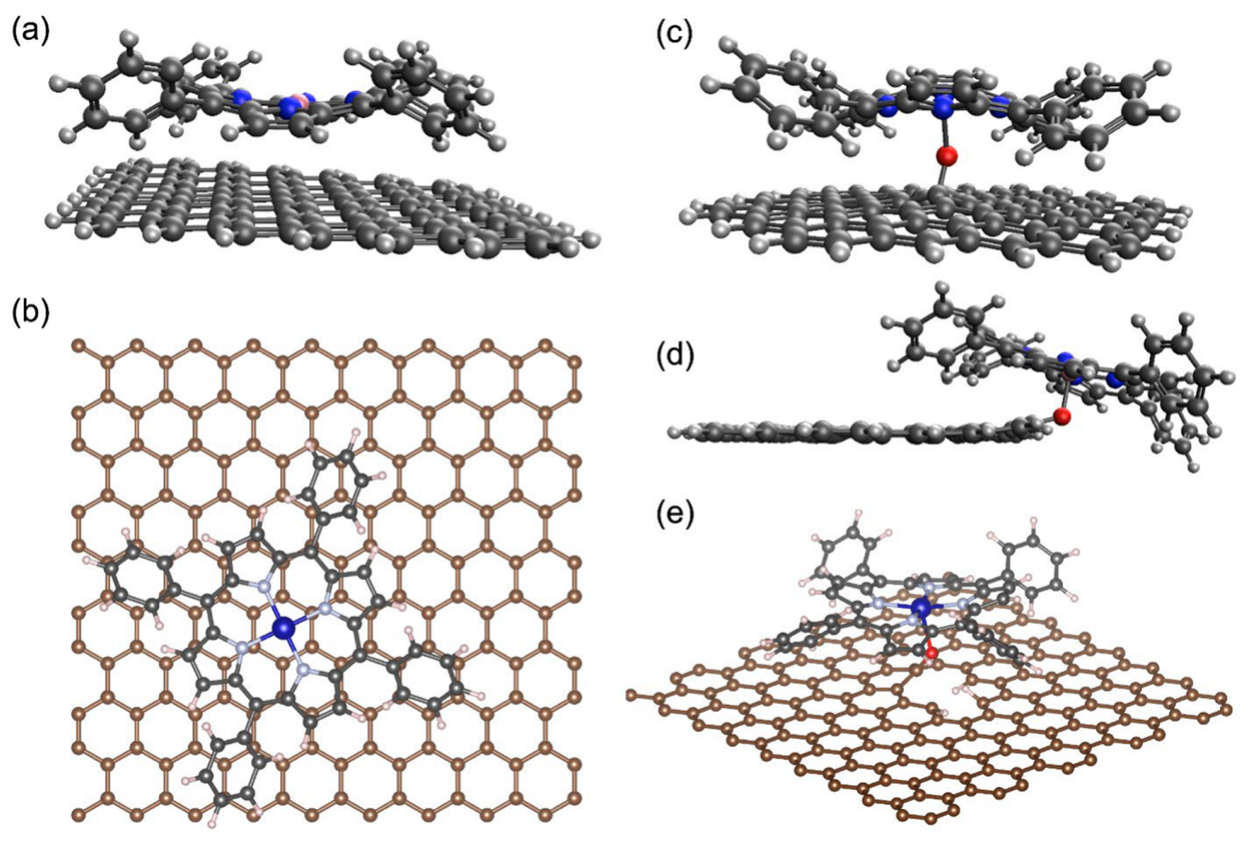
Models of CoTPP-CH studied in this work. (a) CoTPP adsorbed to a H-passivated graphitic flake through π-stacking, referred to as CoTPP/C_96_H_26_. (b) CoTPP adsorbed to a periodic graphene basal plane through π-stacking, referred to as CoTPP/C_periodic_. (c) CoTPP axially ligated to an oxygenated defect on the basal plane of a graphitic flake (denoted CoTPP-O_basal_-C_80_H_22_). (d) CoTPP ligated to an oxygenated defect on the edge plane of a graphitic flake (denoted CoTPP-O_edge_-C_80_H_21_). (e) CoTPP axially ligated to an oxygenated defect on a periodic graphene basal plane (denoted CoTPP-O-C_periodic_).

To explore the PCET chemistry of CoTPP adsorbed to graphitic carbon, we constructed models using both cluster approaches that more accurately treat the adsorbing metalloporphyrin and periodic approaches that accurately treat the extended nature of the surface. The π-stacked cluster-based model was constructed from a basic 96 carbon flake with 26 hydrogens passivating the dangling bonds. The extended surface model was constructed from a rectangular graphene single layer of 180 carbons with periodic boundary conditions. Additional layers of graphene did not significantly affect the Fermi level of the clean surface but increased the density of graphitic states at the Fermi level (Figure S5). The cluster and periodic models are shown with CoTPP adsorbed in Figures 1a and b. For both the cluster-based and periodic approaches, we constructed models with surface oxygenates that can serve as axial ligands for CoTPP. We used a slightly smaller cluster of 80 carbons for the surface oxygenates. As shown in Figures 1c–e, surface oxygenates were placed on the graphitic basal plane or on the graphitic edge. Although it is possible for a water molecule to ligate to the cobalt of CoTPP, we did not perform calculations on such systems because water does not fit on the graphitic side of adsorbed CoTPP and would prevent protonation on the solvent exposed side of CoTPP.

The cluster-based DFT calculations were performed with the Gaussian 16^42^ electronic structure package using the BP86^43,44^ exchange correlation functional and Grimme’s D3 correction.^45^ The BP86 functional has been benchmarked previously for cobalt porphyrin systems.^46,47^ The aqueous environment was described using the conductor-like polarizable continuum model.^48,49^ The periodic DFT calculations were performed with Quantum ESPRESSO^50,51^ using the PBE-D3^45,52^ functional and a Hubbard *U* value^53−56^ of 4 eV on the cobalt atom.^36,57^ The aqueous environment was described using the dielectric continuum model implemented in the Environ module.^58^ Note that polarizable continuum models do not account for explicit hydrogen bonding or solvent structuring, which can be important for reaction thermodynamics at electrochemical interfaces, but are expected to be suitable for the analyses in the present paper.^59^ For the cluster-based models, we explored high and low spin states of the system and selected the lowest-energy state. For the periodic models, we initialized the spin of the system to be low spin, which is the lowest-energy spin configuration. A saddle-shaped distortion accompanied by phenyl group rotation is observed in all of our models of adsorbed CoTPP, consistent with prior atomic force microscopy (AFM) and scanning tunneling microscopy (STM) studies; Raman, UV–vis, and X-ray spectroscopy measurements; and computational studies of immobilized metalloporphyrins.^37,38,60−63^ Additional information on the models and computational details are provided in the Supporting Information.

## Results

We explored ET and PCET reactions in both the cluster-based and periodic model systems. The electrons are assumed to be provided by an external circuit representing the applied electrode potential. Modulation of the applied potential alters the electrode polarization and thus the surface charge. The protons are assumed to be provided by the solution, either directly from water or from a solvated acid molecule. We explored both π-stacked configurations, which are dominated by dispersion interactions, and axially ligated configurations, which involve a chemical bond between the cobalt and a surface oxygenate. Analysis of the molecular orbitals (MOs), projected density of states (PDOS), spin densities, and thermodynamics provides insights into the reaction mechanisms for these different scenarios. Here we define a stepwise PCET mechanism to involve a thermodynamically stable intermediate corresponding to either ET or PT. From a theoretical perspective, such an intermediate would correspond to a minimum on the potential energy surface. For this system, a stepwise mechanism with ET followed by PT would involve a Co(I)TPP intermediate followed by protonation to form Co(III)HTPP. As shown below, we did not find evidence of a Co(I)TPP intermediate but rather observed only concerted PCET mechanisms for all systems studied. This finding is consistent with prior experiments,^22^ indicating that a Co(I)TPP intermediate does not form in aqueous solution because the energy levels of the molecule and electrode shift together upon charging the electrode due to strong electrostatic interactions.

### ET and PCET in π-Stacked Configurations

**Figure 2:**
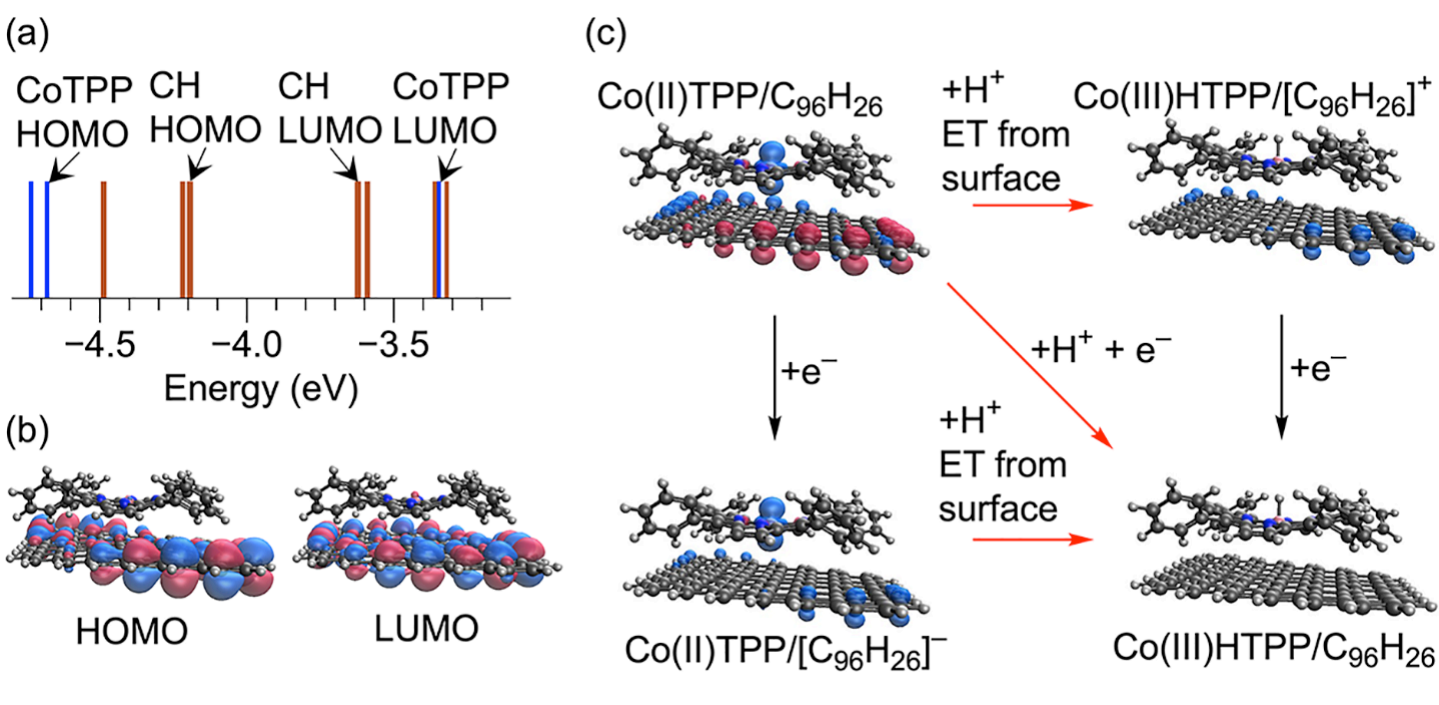
Analysis of adsorption of CoTPP on a graphitic flake. (a) Energy level diagram for CoTPP/C_96_H_26_, where energy levels with C_96_H_26_ centered MOs are plotted in brown and CoTPP centered MOs are in blue. Note that the CoTPP HOMO is singly occupied and corresponds to the alpha spin , whereas the CoTPP LUMO corresponds to the beta spin . (b) HOMO and LUMO for Co(II)TPP/C_96_H_26_. Both are localized on the graphitic flake. (c) Nonstandard square scheme for PCET at CoTPP/C_96_H_26_. All three red arrows indicate concerted PCET, where PT from solution is accompanied by ET from the graphitic surface to CoTPP, forming Co(III)HTPP. Addition of an electron (black arrows) corresponds to ET from the external circuit, which charges the graphitic surface. At constant potential, the electron abstracted from the surface to the CoTPP upon protonation is replaced by an electron from the external circuit (red diagonal arrow). Spin density isosurfaces for Co(II)TPP/C_96_H_26_ (left species) indicate a singly occupied . Formation of the Co–H bond via concerted PCET results in Co(III)HTPP with no unpaired spins (right species). All isosurfaces are plotted with a value of 0.05 Å^–3^.

Before adsorbing on a graphitic surface, CoTPP is expected to be in the Co(II) oxidation state with a single unpaired electron in the  orbital, denoted Co(II)TPP. The singly occupied nature of this d-orbital is reflected by the spin density on the cobalt porphyrin, which is centered on the cobalt and exhibits strong  character. Adsorption onto a graphitic flake through π–π interactions does not result in any chemical bond formation and does not alter the oxidation state of the cobalt through partial charge transfer, as indicated by the spin density remaining largely unaltered compared to the isolated molecule. Adsorption onto the flake, however, does change the nature of the frontier orbitals by introducing graphitic electronic states within the gap between the highest occupied molecular orbital (HOMO) and the lowest unoccupied molecular orbital (LUMO) of CoTPP (Figure 2a). As a result, the HOMO and LUMO of the system composed of CoTPP adsorbed onto the graphitic flake are centered on the graphitic flake (Figure 2b). There is negligible mixing of the orbitals associated with the graphitic flake and CoTPP, indicating relatively weak electronic coupling between the two components.

For the cluster-based model, addition of an electron resulted in reduction of the graphitic flake rather than reduction of Co(II)TPP/C_96_H_26_ to Co(I)TPP/C_96_H_26_. The energy level diagram indicates that the C_96_H_26_ LUMO will be occupied before the CoTPP LUMO (Figure 2a). Charging of the graphitic surface is indicated by a change in the spin density located at the edges of the graphitic flake (Figure 2c, bottom left). PCET occurs at Co(II)TPP/C_96_H_26_ by addition of a proton and an electron to the adsorbed CoTPP and results in the formation of a cobalt hydride, Co(III)HTPP/C_96_H_26_, with no unpaired spins. As mentioned above, the electron is provided by the external circuit, and the proton is provided by the solution. The change in free energy for producing Co(III)HTPP/C_96_H_26_ from Co(II)TPP/C_96_H_26_, a proton, and an electron is only 0.14 eV higher than the analogous process for producing the isolated Co(III)HTPP in solution, corresponding to a more negative proton-coupled redox potential. This similarity in the proton-coupled redox potential for the isolated and adsorbed CoTPP is not unexpected, as the active cobalt center is only weakly interacting with the graphitic flake, and the coordination environment is not modified. In the absence of forming Co(I)TPP/C_96_H_26_, which is thermodynamically unfavorable, the electron must be taken from the graphitic flake simultaneously with protonation of the cobalt, representing a concerted PCET process.

The formation of Co(III)HTPP/C_96_H_26_ through concerted PCET can be represented as a nonstandard square scheme (Figure 2c). All three steps involving protons (red arrows) correspond to concerted PCET due to the absence of Co(I)TPP. As discussed above, adding an electron to Co(II)TPP/C_96_H_26_ charges the surface to yield Co(II)TPP/[C_96_H_26_]^−^ (left black arrow in Figure 2c). Subsequent protonation is accompanied by electron abstraction from the graphitic surface to form Co(III)HTPP via concerted PCET (lower red arrow). In the absence of excess electronic charge on the graphitic surface, protonation of the cobalt is still accompanied by electron abstraction from the graphitic surface to form Co(III)HTPP and a positively charged graphitic surface (upper red arrow). Subsequent addition of an electron neutralizes the graphitic surface (right black arrow). In both cases, the transfer of electronic charge from the graphitic flake to form the Co(III)H is indicated by a total loss of spin density on the adsorbed CoTPP. At constant potential, protonation from solution is accompanied by addition of an electron from the external circuit to ensure that the graphitic state occupancy is unchanged as an electron is abstracted from the surface to form the Co(III)H (diagonal red arrow).

**Figure 3:**
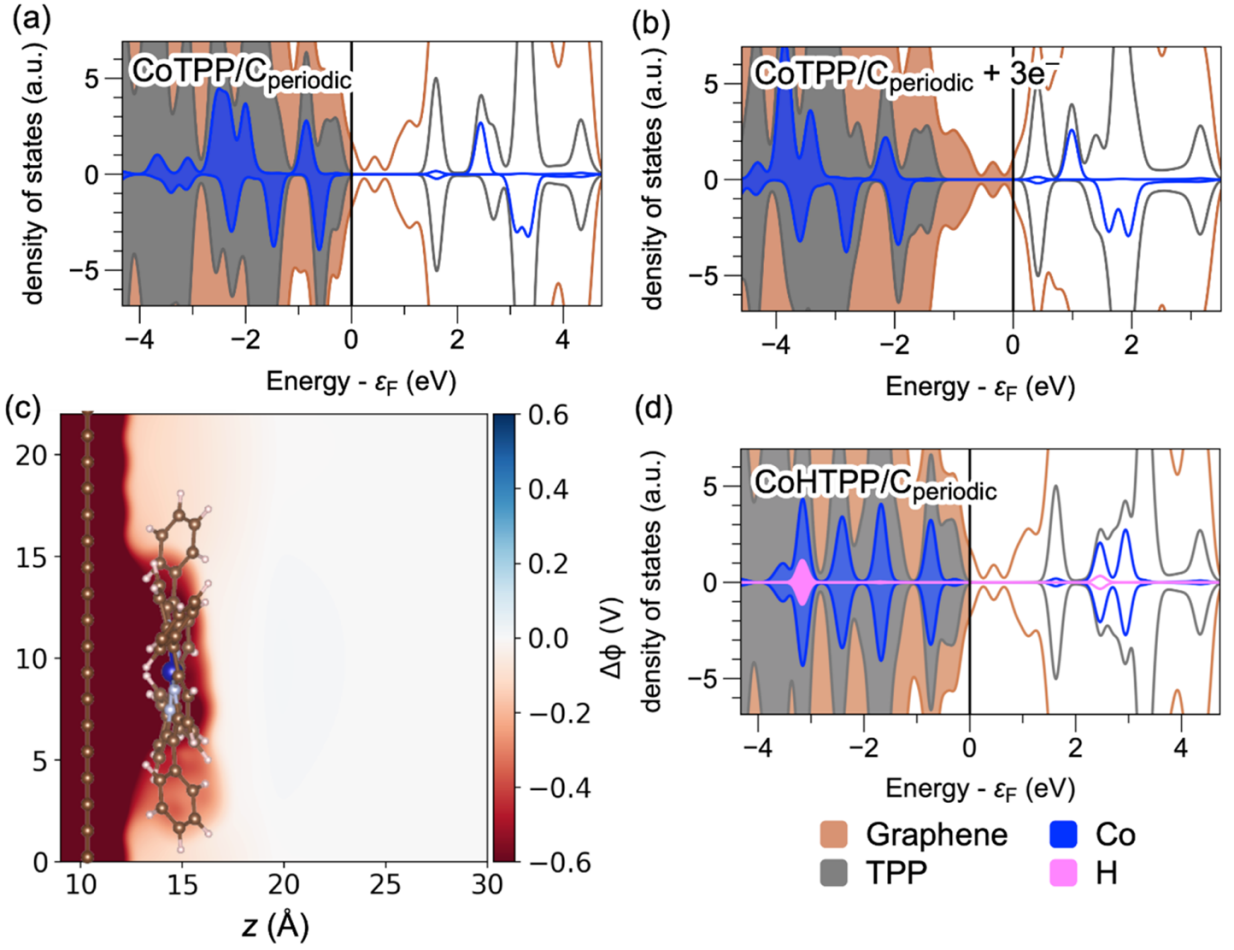
PDOS for CoTPP/C_periodic_, which is CoTPP adsorbed on a periodic graphite plane (a) of neutral charge and (b) with three electrons added, decreasing the electrode potential by ∼0.7 V. (c) Change in the electrostatic potential when three electrons are added to the CoTPP/C_periodic_ system. The electrostatic potential in the bulk solvent region does not change. (d) PDOS for the neutral CoHTPP/C_periodic_ system showing the Co–H bonding state ∼3 eV below the Fermi level and the antibonding state ∼2.5 eV above the Fermi level. The Co(II)-related states in (a) and (b) exhibit energetic splitting between α and β spins (i.e., spin polarization) that is not present for the Co(III)H-related states in (d), where all spins are paired. The position of the  β state above the Fermi level maintains the unpaired spin on CoTPP upon adsorption onto graphene. Integrating the DOS confirmed the surface charging in (b) and the formation of the Co(III)H in (d) (Figures S6 and S7). Figure S11 projects the DOS for (a), (b), and (d) onto the specific cobalt d-states. The color key identifies the electronic states associated with each type of atom.

The discrete electronic states of the cluster-based systems do not fully capture the behavior of an extended surface, whereas periodic models are able to describe delocalized band states of an extended surface. Similar to the graphitic flake systems, adsorption on the periodic graphene basal plane does not result in substantial alteration of the Co(II)TPP electronic structure. The weak electronic coupling between the molecule and the surface is illustrated by the PDOS, which is effectively a superposition of the PDOS for isolated graphene and isolated Co(II)TPP with slight broadening of the CoTPP-related states (Figure S5). The lack of chemical bonding between the molecule and the surface is also consistent with previous work on metallophthalocyanines adsorbed on graphene.^30^ In the presence of an extended surface and the band-structure limit, adding electrons to the system only results in the occupation of graphitic electronic states and charging of the surface and not the formation of a Co(I)TPP species (Figures 3a and b). The change in total surface charge of 3e^–^ corresponds to a shift of −0.70 V in the applied potential, changing the potential from 0.26 V to −0.44 V vs SHE, which is similar to the potential at which HER is observed experimentally (ca. −0.50 V vs RHE). Although the addition of more electrons may eventually allow formation of Co(I) in this model system, the high density of graphitic states would prevent this reduction of cobalt at potentials relevant to HER in experimental systems.

The charging of the electrode surface, however, does change the electrostatic potential at the Co(II)TPP through electrostatic coupling (Figure 3c). The polarization of the electrode and Co(II)TPP is expected to attract proton donors to the adsorbed Co(II)TPP to facilitate PCET. The separation between the adsorbed CoTPP and the graphitic surface is ∼3.4 Å, which is likely too narrow for insertion of a water molecule. Moreover, both CoTPP and graphene are hydrophobic, inhibiting the formation of structured water at the interface. Thus, the adsorbed CoTPP and graphitic surface can be viewed as cosolvated, and the CoTPP experiences nearly the same electrostatic potential as the electrode surface. This behavior attenuates as the distance between the Co(II)TPP and surface is increased, to a point where the solvated Co(II)TPP does not experience the same electrostatic potential as the electrode surface. Specifically, increasing the separation of the CoTPP from the surface to ∼6.5 Å leads to negligible electrostatic coupling between the CoTPP and the surface, mainly because solvent occupies the region between them (Figure S11).

The distinction between electronic coupling and electrostatic coupling is important in this analysis. Electronic coupling for these systems is reflected by the influence of the graphitic surface on the CoTPP electronic structure and vice versa. Electronic coupling is expected to be relatively weak for π–π interactions, although a slight broadening of the CoTPP-related states in the PDOS indicates a small degree of electronic coupling. Electrostatic coupling for these systems is reflected by the similarity in electrostatic potential at the adsorbed molecule and the surface. Although there may be a small electrostatic potential drop between the surface and the molecule, the molecule and surface experience nearly the same electrostatic potential and polarization because the molecule is cosolvated with the electrode. This explanation is in line with the experimental observation that moving from aqueous to acetonitrile electrolyte, where the molecule is better solvated, serves to eliminate this electrostatic coupling and leads to classical mediation via Co(II/I) redox. In aqueous media, the molecule is not screened from the charge on the surface, and this strong electrostatic coupling causes the majority of the electrostatic potential drop to occur between the molecule and solution rather than between the electrode and the molecule (Figure 3c). Charging of the electrode does not lead to significant accumulation of electron density at the Co center due to the high density of graphitic states and relatively weak electronic coupling. However, the molecule is polarized by the charged electrode, thereby attracting protons to facilitate concerted PCET.

Adding a proton and an electron to the Co(II)TPP/C_periodic_ system produces the Co(III)HTPP/C_periodic_ species (Figure 3d). As for the graphitic flake, this concerted PCET reaction involves electron abstraction from the graphitic surface to CoTPP upon protonation. Under constant potential conditions, abstraction of charge from the surface to populate CoTPP frontier orbitals upon protonation requires the addition of an electron from the external circuit to maintain the occupation of the graphitic states.^64,65^ Moreover, the bonding and antibonding orbitals created by the interaction of the Co  orbital and the proton are visible in the PDOS of the CoHTPP/C_periodic_ system, ∼3 eV below and ∼2.5 eV above the Fermi level, respectively (pink in Figure 3d). This observation further supports the chemistry suggested by the graphitic cluster models, as an electron from the surface is required to populate this new bonding orbital formed by the interaction between Co and the proton. For CoTPP adsorbed by π–π interactions, the concerted PCET mechanism implies that protonation is accompanied by ET from the graphitic surface and that this ET process will not occur in the absence of protonation.

### Effects of Axial Ligation to a Surface Oxygenate

**Figure 4:**
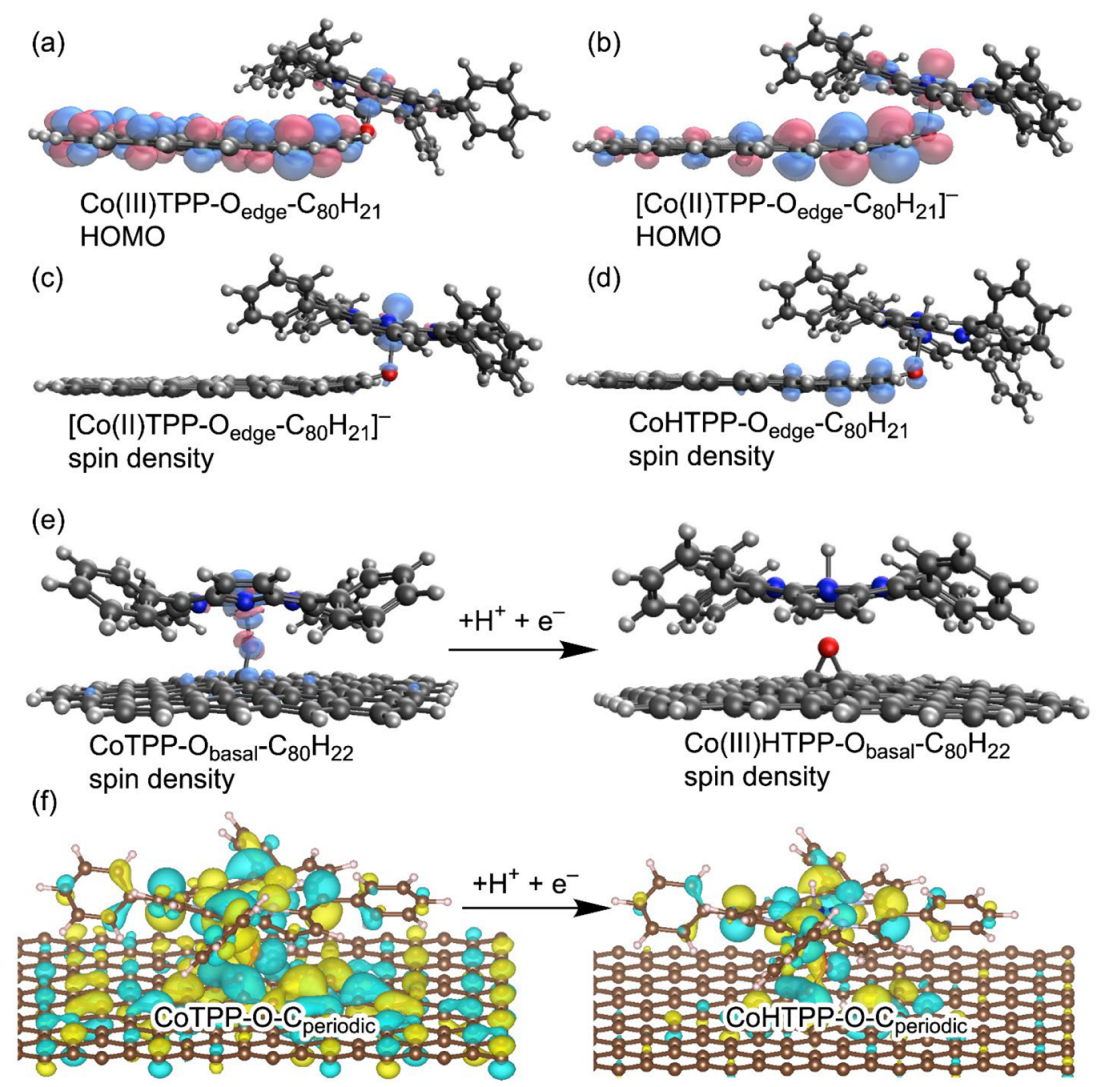
MOs and spin densities for ET and PCET in systems where CoTPP axially ligates to a surface oxygenate in both cluster and periodic systems. For ligation to an oxygenate on the edge of a graphitic flake, the HOMOs show mixed graphitic and CoTPP character for (a) Co(III)TPP-O_edge_-C_80_H_21_ and (b) the negatively charged [Co(II)TPP-O_edge_-C_80_H_21_]^−^, formed by addition of one electron to Co(III)TPP-O_edge_-C_80_H_21_. Spin densities for this type of ligation are localized on the cobalt for (c) [Co(II)TPP-O_edge_-C_80_H_21_]^−^ and primarily on the graphitic flake for (d) CoHTPP-O_edge_-C_80_H_21_. (e) Addition of a proton and electron to the neutral CoTPP-O_basal_-C_80_H_22_, where the surface oxygenate is on the graphitic basal plane. Formation of Co(III)HTPP results in the breaking of the Co–O bond. (f) HOMOs for the periodic graphene models with axial ligation before adding a proton and electron, CoTPP-O-C_periodic_ (left), and after adding a proton and electron, CoHTPP-O-C_periodic_ (right). In this case, HOMO refers to the contribution of the wave function to the electron density for the highest occupied electronic state.

Oxygen containing functional groups, which are native to graphitic surfaces and highly likely on edge planes, can serve to enhance electronic coupling between CoTPP and the graphitic surface relative to the π-stacking case. The formation of a direct chemical bond between the adsorbing electrocatalyst and a graphitic surface can affect the oxidation state of the cobalt. Ligation to an edge-hosted oxygen (i.e., CoTPP-O_edge_-C_80_H_21_) alters the oxidation state from Co(II)TPP to Co(III)TPP with bond formation between the cobalt and oxygen. The altered oxidation state is suggested by the lack of spin density with  character in the charge neutral state. In the neutral state, the HOMO is mixed between the graphitic flake and the CoTPP (Figure 4a). After the addition of one electron, the HOMO remains partially delocalized over the flake (Figure 4b), but a spin density reminiscent of Co(II)TPP with  character is recovered (Figure 4c).

Different modes of axial ligation result in other effects on the adsorbed CoTPP oxidation state. For example, ligation of Co(II)TPP to an oxygenate on the graphitic basal plane in both the cluster (CoTPP-O_basal_-C_80_H_22_) and periodic (CoTPP-O-C_periodic_) models does not result in the formation of a Co(III) species. In this case, ligation of the cobalt center results in mixing of graphitic and CoTPP orbitals and partial charge transfer with the graphitic surface. This partial charge transfer is illustrated by the spin density distributed across the cobalt, oxygen, and proximal carbons (Figure 4e, left), leading to a more ambiguous cobalt oxidation state. Similar to the π-stacked systems, surface charging enabled by electron flow from the external circuit does not reduce CoTPP to a Co(I) oxidation state (Figure S8). As for the edge-ligated case, axial ligation to a periodic slab exhibits electronic charge delocalization upon the addition of electrons.

When CoTPP is ligated to a surface oxygenate, either on the edge or the basal plane, PCET to the system leads to Co(III)HTPP. Following PCET at the edge ligated CoTPP, the added electron is delocalized across the CoHTPP and graphitic surface, resulting in nonzero spin density that is primarily located on the graphitic flake (Figure 4d). For ligation to a basal plane oxidic defect, PCET to the system results in the breaking of the Co–O bond during formation of the Co(III)H state. Thus, formation of the Co(III)H likely involves abstracting electronic charge from the surface through the Co–O bond and breaking the Co–O bond, leaving the Co(III)HTPP stabilized on the surface by dispersion interactions (Figure 4e). The formation of this Co–H bond is 0.27 eV more thermodynamically favorable compared to the analogous formation of isolated Co(III)HTPP in solution, corresponding to a slightly less negative proton-coupled redox potential. For the edge-ligated system, the Co–O bond is not broken during PCET, but the ET is still likely to occur through this bond based on delocalization of the HOMO in the negatively charged [Co(II)TPP-O_edge_-C_80_H_21_]^−^ (Figure 4b). Such behavior reflects the direct electronic coupling provided by the chemical bond connecting the CoTPP and the surface. This strong electronic coupling and associated delocalized charge transfer indicate that the adsorbed CoTPP should be viewed as part of the graphitic surface in this configuration. This behavior is markedly different from the π-stacked systems, where CoTPP is electronically distinct from the graphitic surface.

The periodic model of CoTPP axially ligating to an oxidic defect shows very similar behavior as the cluster graphitic-flake models, with MOs delocalized over both the surface and the CoTPP (Figure 4f) and similar delocalization of the cobalt spin over the oxygen and carbon (Figure S10). Both cluster and periodic models reflect that when CoTPP is axially ligated to a surface defect, it is directly electronically coupled to the surface. Interestingly, this behavior is reminiscent of GCC systems with organic acids attached to the graphitic surface by a phenazine bridge, where strong electronic coupling is also enabled by chemical bonding.^24^

### Generalized Mechanistic Model and Relationship to Experiment

**Figure 5:**
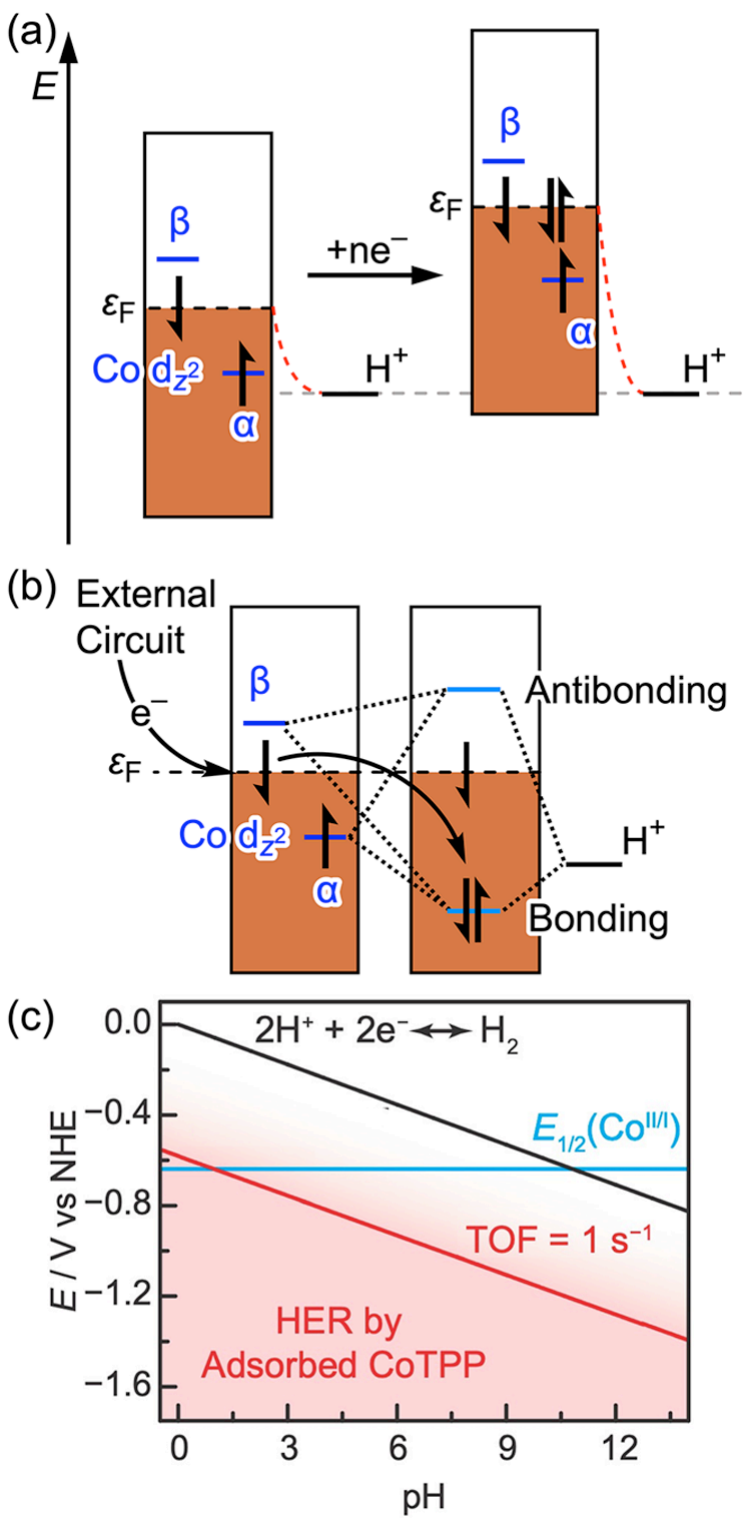
(a) Energy diagram showing how the energy levels of the system change upon charging of the electrode. Electrons add to the Fermi level (ε_F_) but do not populate the β spin state of the Co  orbital, which is above the Fermi level, preventing Co(II/I) redox. Electrode charging increases the electrostatic potential drop at the interface (red dotted line), increasing the driving force for proton transfer to the interface and thereby enabling catalysis across the pH range. (b) Molecular orbital diagram for the formation of a Co–H bond at CoTPP adsorbed on graphitic carbon by concerted PCET. Due to spin polarization, the Co(II)  α state is below the Fermi level, whereas the Co(II)  β state is above the Fermi level. As the proton approaches the Co active site, bonding and antibonding orbitals are formed due to the interaction of the Co  α and β spin orbitals, which correspond to the same spatial orbital, with the hydrogen 1s orbital. The bonding and antibonding states are created by mixing the Co  and hydrogen 1s orbitals. As the bonding orbital drops below the Fermi level (ε_F_), it becomes occupied by one electron from the singly occupied Co  and one electron abstracted from the graphitic states at ε_F_. The electron abstracted from the graphitic states is replaced by an electron from the external circuit. Before PCET the Co  is spin-polarized, and after PCET the Co–H bonding orbital is unpolarized (see Figures 3a and d and Figure S10). (c) CoTPP catalyzes HER across the entire pH range (red) even at pH values where the Co(II/I) redox (blue) is at an underpotential to HER (black). Part (c) adapted with permission from ref (22). Copyright 2022 Springer Nature.

Although the π-stacking and axial ligation modes of adsorption lead to distinct PCET thermochemistry and differing degrees of electronic coupling, the mechanistic picture for concerted PCET is conserved across both adsorption modes. Charging of the electrode polarizes the CoTPP as well as the interface and thereby attracts protons, enabling PCET at the adsorbed CoTPP (Figure 5a). As the proton approaches the cobalt active site, interaction of the Co  orbital with the H 1s orbital leads to the formation of a new pair of Co–H bonding and antibonding orbitals (Figure 5b). As the Co–H bonding orbital drops below the Fermi level, an electron abstracted from the Fermi level to occupy this Co–H bonding orbital is subsequently replaced by an electron from the external circuit to maintain constant electrode potential. This mechanism is consistent with the model put forward by Hoffmann for thermal reactions between molecules and surfaces.^64^ Importantly, even in the limit of relatively weak electronic coupling for the π-stacked mode of adsorption, the molecule still experiences the interfacial electrostatic potential. Thus, irrespective of the adsorption mode, electrode charging adds electrons to the Fermi level, and the resulting interfacial electrostatic potential drop shifts all the states in tandem rather than changing the electron occupancy of the Co quasi-molecular d-states, thereby excluding Co(II/I) redox (Figure 5a). As a consequence, a redox wave for the Co(II/I) process is not observed experimentally for the adsorbed CoTPP in aqueous media.^22^ This phenomenon is also the reason that the adsorbed CoTPP carries out HER catalysis across the entire pH range (Figure 5c, red), even at pH values where the Co(II/I) redox potential of a water-soluble molecular analogue lies at an underpotential to the overall reaction (Figure 5c, blue vs black). Since electrode polarization augments the attraction of the protons to the surface, rather than filling the Co d-states, a sufficiently negative potential can drive HER in alkaline media via the same concerted PCET mechanism with band to bond electron redistribution.

## Conclusions

The immobilization of a cobalt porphyrin on a graphitic surface inherently alters the mechanism of PCET. In this work, we show that electrostatic coupling between CoTPP and a graphitic surface leads to a concerted PCET mechanism for formation of Co(III)HTPP that bypasses the Co(I) state. In π-stacked models dominated by dispersion interactions between the CoTPP and the graphitic surface, addition of electrons charges the graphite surface rather than reducing the cobalt, and concerted PCET occurs by electron abstraction from the surface upon protonation of the CoTPP. Axial ligation of the CoTPP to surface oxygenates results in a more ambiguous cobalt oxidation state and engenders stronger, through-bond electronic coupling, but the fundamental concerted PCET mechanism is the same as that for the π-stacked models. Regardless of the adsorption mode, both cluster and periodic extended surface models show that Co(II/I) redox is circumvented in the concerted PCET mechanism. The concerted PCET mechanism implicated by these models is that the cobalt d-state localized quasi-molecular orbital interacts with a proton from solution and an electron from the delocalized graphitic band states to produce a Co–H bonding orbital below the Fermi level. This work has broad implications for how we understand and harness reactions occurring at surface immobilized catalysts, where electrostatic coupling between the surface and the molecule is able to fundamentally change the PCET mechanism.

## Supporting Information Available

Construction of model systems, additional computational details and calculations, free energy calculations, Mulliken spin populations, Co oxidation state analyses, projected DOS and integrated DOS, additional spin densities, and additional electrostatic potential profiles (PDF)
