# Supplementary material for: Concerted Proton-Coupled Electron Transfer to a Graphite Adsorbed Metalloporphyrin Occurs by Band to Bond Electron Redistribution
Source: ACS Cent Sci. 2023 Apr 19;9(5):927–36. doi: 10.1021/acscentsci.3c00186 (PMC10214502; doi:10.1021/acscentsci.3c00186)
Supplement: Supplementary file 1 — oc3c00186_si_001.pdf [file oc3c00186_si_001.pdf]

Supporting Information

Concerted PCET to a Graphite Adsorbed Metalloporphyrin Occurs by Band to Bond Electron Redistribution

Phillips Hutchison[1], Corey J. Kaminsky[2], Yogesh Surendranath[2], and Sharon Hammes-Schiffer[1]*

[1]*Department of Chemistry, Yale University, New Haven, CT 06520, United States*

[2] *Department of Chemistry, Massachusetts Institute of Technology, Cambridge, MA 02139, United States*

*email: sharon.hammes-schiffer@yale.edu

Table of Contents

Construction of Graphitic Surface Models ***S2***
Additional Computational Details ***S3***
Impact of Aliphatic Linker ***S4***
Calculation of Relative Free Energy of Co-H Bond Formation ***S5***
Graphitic Cluster Size and Electronic States ***S6***
Impact of Multiple Carbon Layers ***S7***
Projected Density of States and Integrated DOS ***S8***
Density of States for Periodic Models with Axial Ligation ***S11***
CoTPP Spin Density in Periodic Models ***S13***
Cobalt Oxidation State and d-States ***S13***
Decay of Electrostatic Potential between CoTPP and Graphene ***S16***

Construction of Graphitic Surface Models

The graphitic clusters were constructed from multiple fused aromatic carbon rings. For the π-stacked systems, we constructed two flakes with different stoichiometries. The smaller cluster consisted of 80 aromatic carbons, and the larger cluster (discussed in the main text) consisted of 96 aromatic carbons, as shown in Figures S1a and S1b, respectively. For both π-stacked flake models, all dangling bonds were passivated with hydrogens. The results from the larger flake are presented in the main text. Two different surface oxygenates were constructed on the smaller flake. The first oxygenate (Figure S1c) was placed on the graphitic edge by replacing a single hydrogen with an oxygen atom. The second oxygenate (Figure S1d) was constructed on the basal plane, slightly offset from the center.

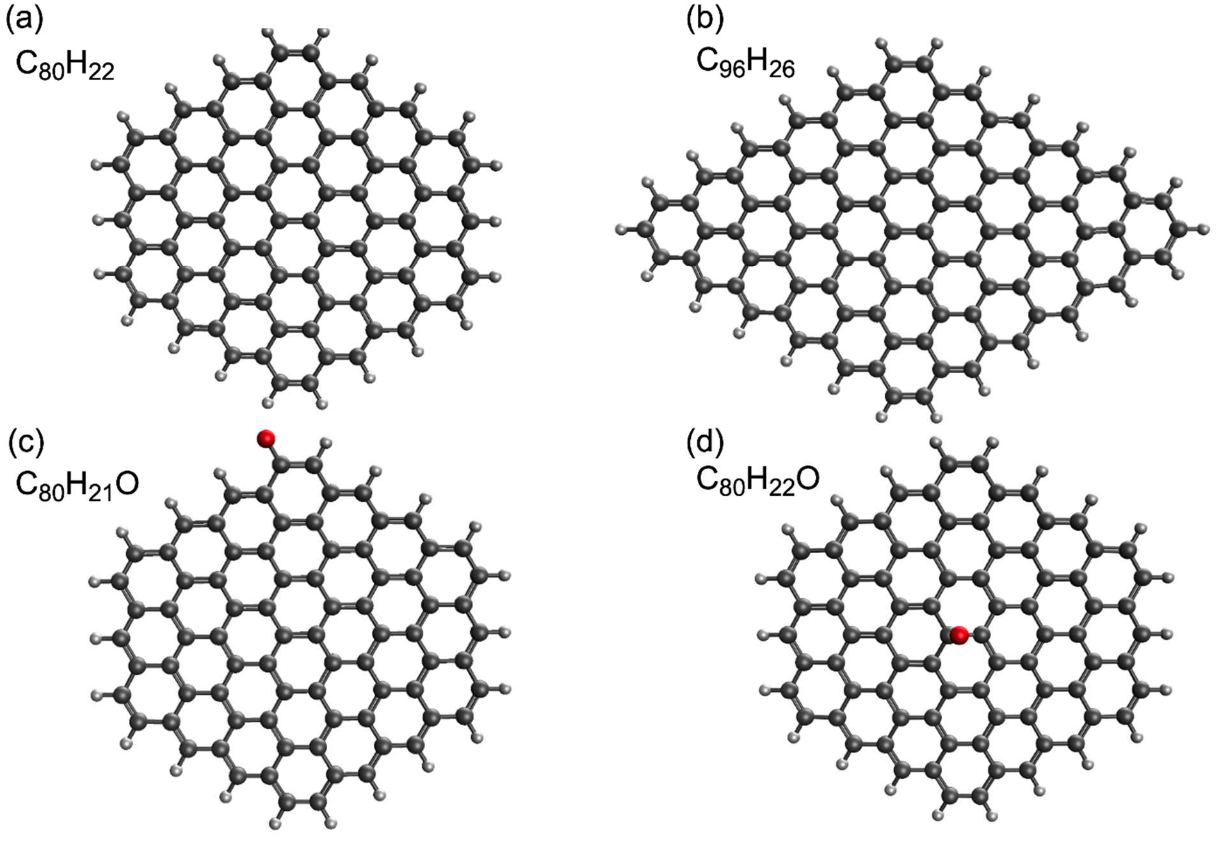

Figure S1. Graphitic flake models considered in this work with stoichiometries of (a) $C_{80}H_{22}$ (b) $C_{96}H_{26}$ (c) $C_{80}H_{21}O$ (d) $C_{80}H_{22}O$.

The periodic graphite models are based on a single-layer graphene sheet consisting of 180 carbon atoms ($C_{periodic}$). The graphene sheet was constructed as a $9 \times 5 \times 1$ rectangular supercell

such that the adsorbed CoTPP is isolated from its own periodic images by at least 6.2 Å. The lattice parameters for the graphene sheet were taken from the optimized lattice parameters for bulk graphitic carbon. Calculations for bulk carbon were conducted with a $16 \times 16 \times 16$ k-point mesh. The unit cell dimensions for all periodic surface models were 21.189 Å × 21.375 Å × 41.980 Å. To construct the oxygenate on the basal plane, a pore was created by removing two carbons from the lattice. One of the dangling carbon bonds was then passivated with an oxygen, while the remaining three dangling bonds were passivated with hydrogens. This was necessary to avoid the formation of a bond between the oxygen and two carbons of the graphitic surface. Both periodic models are shown in Figure S2.

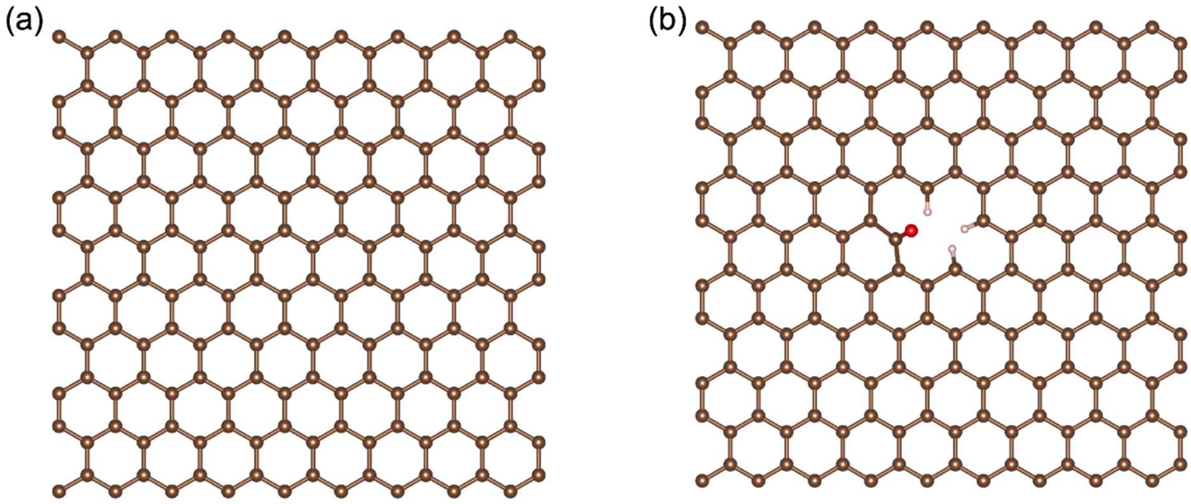

Figure S2. Periodic graphitic surface models based on (a) a pristine graphene sheet, $C_{periodic}$, and (b) a single oxygenate on the basal plane, O-$C_{periodic}$.

Additional Computational Details

All calculations for the graphitic flake models were conducted using the Gaussian16 package[1] with parameters described in the main text. For the π-stacked models, the geometry of Co(II)TPP, which is charge neutral, was fully optimized prior to being positioned above the graphitic flake, and then the entire system was geometry optimized at zero total charge. Further reductions of the neutral system were conducted as single-point energy calculations, but systems with an added proton and electron was geometry optimized. Graphitic flake models with CoTPP axially ligated to either basal or edge plane oxygenates were treated in a similar manner but were

allowed to fully geometry optimize when reduced from the charge neutral state. In these calculations, the 6-31G(d,p) basis set was used for carbon, hydrogen, nitrogen, and oxygen, and the LANL2DZ effective core potential[2-10] was used for cobalt. These calculations were performed in implicit solvent using the conductor-like polarizable continuum model (C-PCM) and the dielectric constant of water.[11, 12] The stationary points were confirmed to be minima with frequency calculations.

As mentioned above, the geometry for the periodic graphene plane was obtained from the lattice constants for bulk graphitic carbon. For the periodic models with a pristine graphene plane, the surface was frozen during geometry optimizations. With the surface oxygenate, the hydrogen and oxygen atoms of the pore, as well as the carbon bonded to the oxygen, were optimized. In systems containing CoTPP or CoHTPP, geometry optimizations were conducted for only the charge neutral systems at the gamma-point. The forces in geometry optimizations were converged to 10^{-3} Ry/Å, and the total energies were converged to 10^{-4} Ry. The optimized geometries were then used in single-point energy calculations with a denser 3×4×1 k-point mesh to better capture the electronic structure of graphene and understand the effects of added charge. Projected augmented wave (PAW) pseudopotentials[13] and Gaussian smearing of 0.05 Ry were used. These periodic DFT calculations were performed with Quantum ESPRESSO[14, 15] in polarizable continuum solvent with the dielectric constant of bulk water ($\varepsilon = 78.3$) using the Environ module.[16] For calculations performed with added electrons, the net charge of the atomistic system was neutralized by placing a 1 Å wide strip of opposite charge in the bulk solvent. When the CoTPP was adsorbed, this counter charge was centered at $z = 20$ Å such that the counter charge did not intersect the electron density of the adsorbed CoTPP. When the CoTPP was moved 3.1 Å further away from the surface, the counter charge was moved to $z = 14$ Å or $z = 25$ Å. Periodic boundary conditions were disabled in the *z*-direction, and the parabolic correction for the periodic boundary conditions was employed. Free energies were not calculated for the periodic systems.

Impact of Aliphatic Linker

To understand the impact of the aliphatic linkage that tethers CoTPP to the graphitic surface in the motivating experiments, we geometry optimized CoTPP attached to the $C_{80}H_{22}$ cluster through the aliphatic linkage. The optimized geometry was not dissimilar to those obtained through the π-stacked models, with deviations originating from the folding of the aliphatic linker. In the model

with the aliphatic linker, the cobalt is positioned 3.49 Å from the nearest carbon, whereas the cobalt is positioned 3.46 Å from the nearest carbon in the π-stacked models without the linker. The optimized geometry of the model with the aliphatic linker is shown in Figure S3. Additionally, the linker does not affect the frontier orbitals of the composite system, in that the HOMO for the charge neutral system is consistent with the Co(II)TPP/$C_{80}H_{22}$ results. These combined results suggest that it is reasonable to omit the aliphatic linker in computational models exploring the interactions between the graphitic carbon and CoTPP.

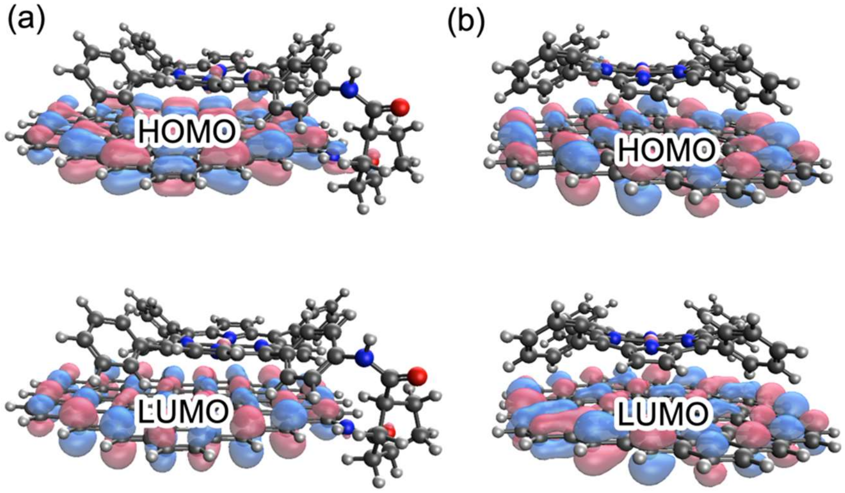

Figure S3. Differences in the frontier orbitals for the Co(II)TPP/$C_{80}H_{22}$ graphitic flake model when (a) CoTPP is tethered by an aliphatic linkage and (b) the tether is omitted.

Calculation of Relative Free Energy of Co-H Bond Formation

The free energy change associated with forming the Co–H bond was determined for the cluster models. This was accomplished by subtracting the free energy of the adsorbed CoTPP species from the adsorbed CoHTPP species. In this process, one electron and one proton are added to the CoTPP species to form the CoHTPP species, and all species are neutral. The resulting free energy change was then calculated relative to the free energy change for forming the Co–H bond in the isolated molecule:

$$\Delta\Delta G_{\mathrm{HAT}} = G(\mathrm{CoHTPP/C}) - G(\mathrm{CoTPP/C}) - G(\mathrm{CoHTPP}) + G(\mathrm{CoTPP}) \qquad \text{(S1)}$$

G(CoHTPP/C) and *G*(CoTPP/C) are the Gibbs free energies of the surface adsorbed Co(III)HTPP and Co(II)TPP, respectively. *G*(CoHTPP) and *G*(CoTPP) are the Gibbs free energies of the isolated Co(III)HTPP and Co(II)TPP molecules, respectively. The Gibbs free energies include corrections for zero-point vibrational energy, entropy, and solvation.

Table S1. Relative Free Energies (eV) for Forming a Co–H Bond in CoTPP Adsorbed on Various Graphitic Carbon Clusters.

System	$\Delta\Delta G_{HAT}{}^{a}$
CoTPP (No Cluster)	0.0000
CoTPP/$C_{80}H_{22}$	–0.1106
CoTPP/$C_{96}H_{26}$	0.1368
CoTPP-O_{edge}-$C_{80}H_{21}$	–0.2688
CoTPP-O_{basal}-$C_{80}H_{22}$	–0.0085

[a]All free energy changes are given relative to the free energy for forming the Co(III)HTPP from Co(II)TPP in C-PCM solvent by adding a proton and an electron.

Graphitic Cluster Size and Electronic States

The size of the graphitic flake used to mimic surface-like behavior has an important effect on the predicted chemistry of adsorbed molecules. In the case of Co(II)TPP adsorbing on graphitic carbon, a graphitic flake should be large enough to introduce graphitic electronic states within the Co(II)TPP HOMO-LUMO gap. The $C_{80}H_{22}$ flake is the smallest size flake studied that introduces a graphitic electronic state within the CoTPP HOMO-LUMO gap. Although the HOMO for the Co(II)TPP/CH system with this flake is centered on the flake, the next two highest electronic states are nearly degenerate and have Co(I)TPP and CH character, respectively (Figure S4a). The larger $C_{96}H_{26}$ flake has many more CH related electronic states within the Co(II)TPP HOMO-LUMO gap, though they remain discrete (Figure S4b). This model is more representative of a physically realistic system and is used for the analysis in the main paper. To converge to the band-structure limit probed by the periodic calculations, much larger graphitic clusters would be needed.

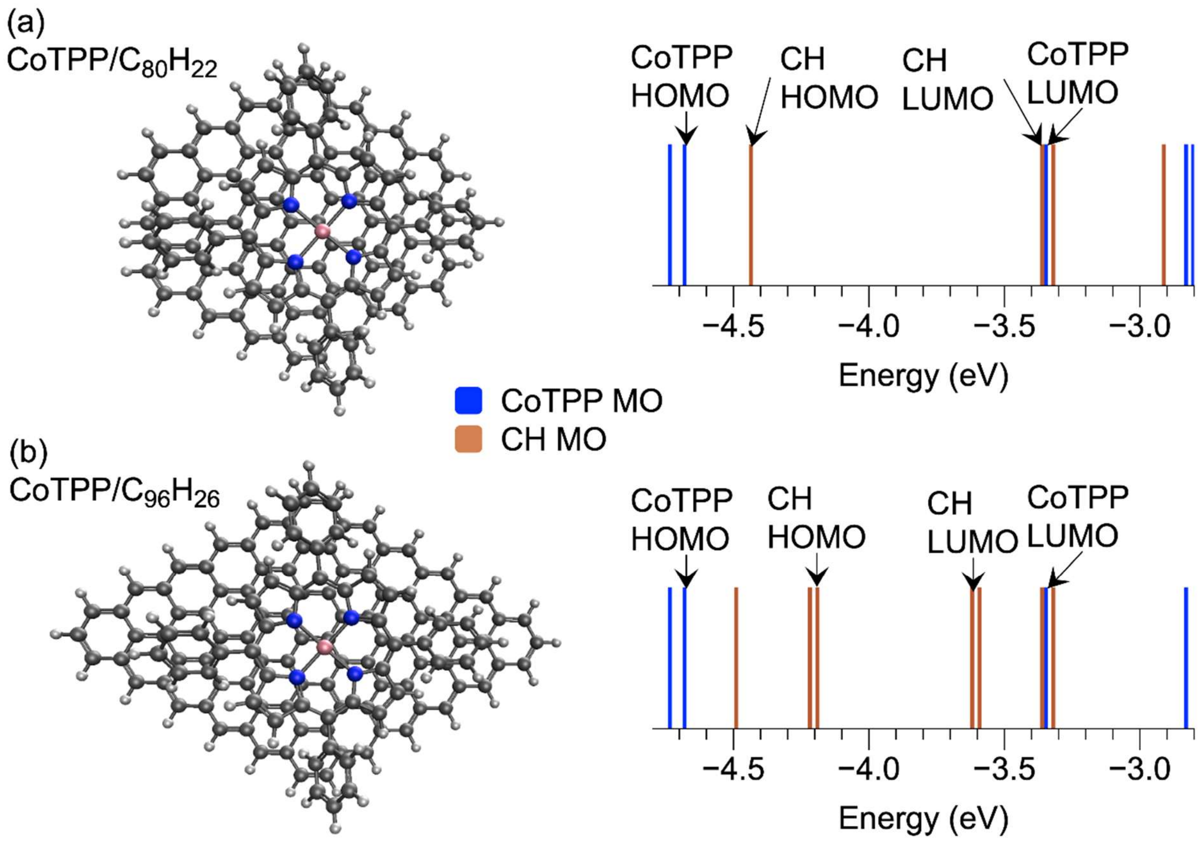

Figure S4. Molecular orbital diagrams for (a) Co(II)TPP/$C_{80}H_{22}$ and (b) Co(II)TPP/$C_{96}H_{26}$ graphitic flake models with CH related states in brown and CoTPP related states in blue. Labels on the MO diagrams denote the HOMO and LUMO centered on the CoTPP and graphitic cluster.

Impact of Multiple Carbon Layers

A more realistic model of the graphitic surface would contain multiple layers of graphitic carbon to mimic the bulk-like nature of the surfaces probed experimentally. To test the impact of multiple graphene layers on our calculated electronic structure, we performed calculations using a smaller graphene model containing 24 atoms per layer for one, two, and three layers. For these calculations, we used a slightly different 12×14×1 k-point mesh than used for the graphene single layer with adsorbed CoTPP. The Fermi energy in each calculation was corrected for the potential shift due to the Gaussian smearing of the nuclei. The Fermi energies obtained from the three different calculations varied by less than 0.01 eV. As expected, the density of states increased as the number of layers increased. The projected density of states (PDOS) for these calculations are given in Figure S5. Given the small changes to the Fermi level, the single layer graphene was determined to be a sufficient model for the surface.

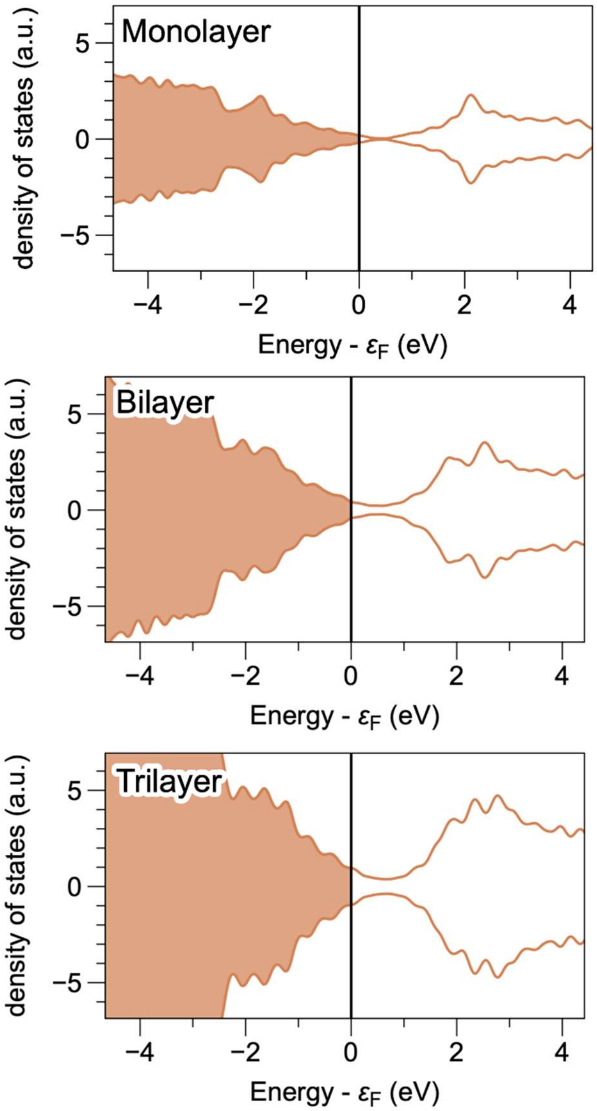

Figure S5. PDOS for periodic systems composed of one, two, and three graphene layers in the absence of CoTPP.

Projected Density of States and Integrated DOS

The PDOS for the periodic systems was obtained by projecting the wavefunction onto an atomic orbital basis. To better isolate the cobalt oxidation state, we separated out the projection onto cobalt states from the projection onto the carbon, nitrogen, and hydrogen states of the porphyrin. Generally, the cobalt states near the Fermi energy (ε_F) and involved in the chemistry of CoTPP are d-states, with the s-states and p-states being lower in energy. The graphene related PDOS is continuous, as shown in the main text, consistent with the literature. The PDOS for the CoTPP/$C_{periodic}$ system can be reasonably well reproduced by adding the PDOS for the isolated

graphene sheet and the PDOS of an isolated CoTPP, both at zero total charge. This superimposable nature reflects the lack of chemical bonding between the CoTPP and the graphene (Figure S6).

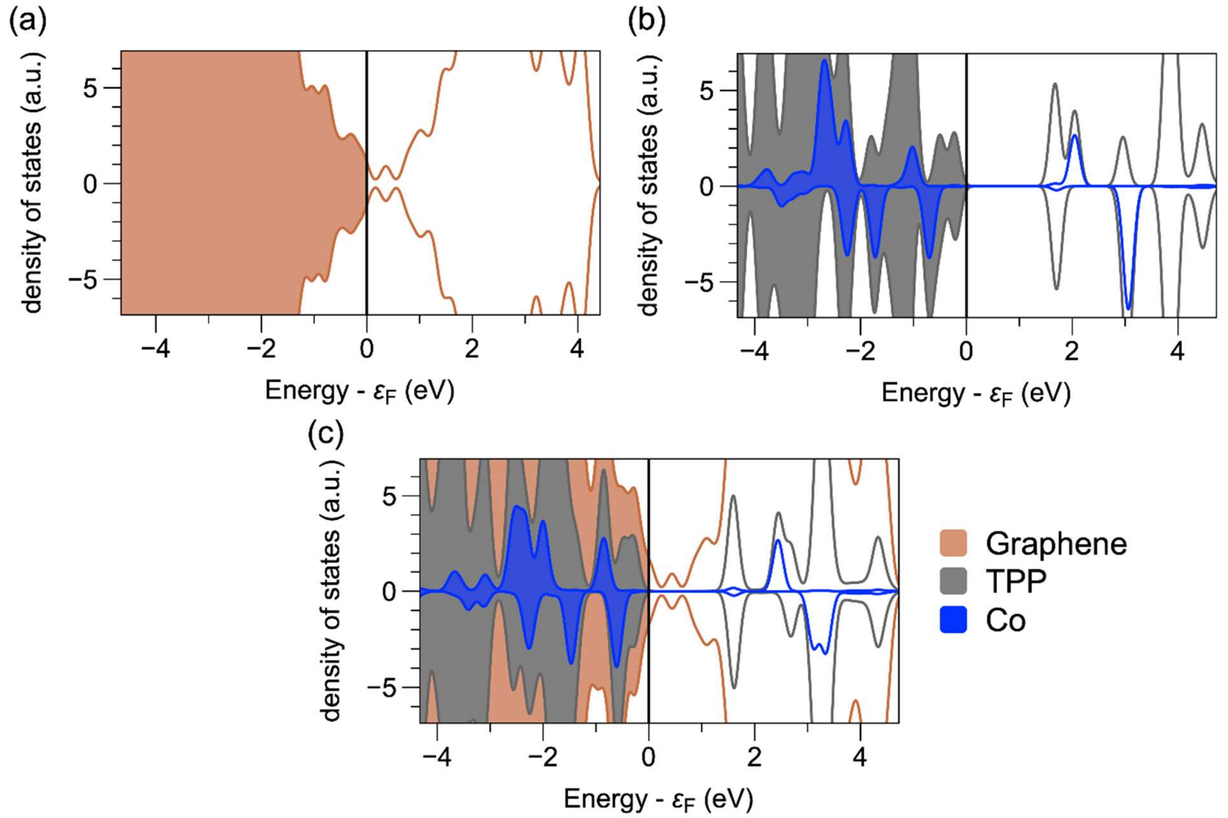

Figure S6. PDOS for periodic systems composed of (a) graphene, (b) CoTPP, and (c) CoTPP π-stacked on graphene (i.e., CoTPP/$C_{periodic}$). The PDOS for the combined system is similar to the sum of the PDOS for the individual systems. The color key identifies the electronic state associated with each component of the system.

We used the integrated DOS to determine how ET and PCET to the system affect the occupancy of specified electronic states. For this purpose, we calculated the value of the integrated DOS at the Fermi level for the Co, porphyrin, and graphene related electronic states before and after ET or PCET. For ET processes associated with surface charging, the occupation of the porphyrin and cobalt related electronic states, indicated by the value of the integrated PDOS at the Fermi energy, is largely unchanged, as shown in Figure S7. This analysis implies that all added electrons occupy graphitic electronic states. For PCET that produces a cobalt hydride, however, the CoTPP related electronic states become occupied by approximately one additional electron, as

shown in Figure S8. In both cases, redistribution of electronic charge will lead to non-integer changes in the occupancy of electronic states.

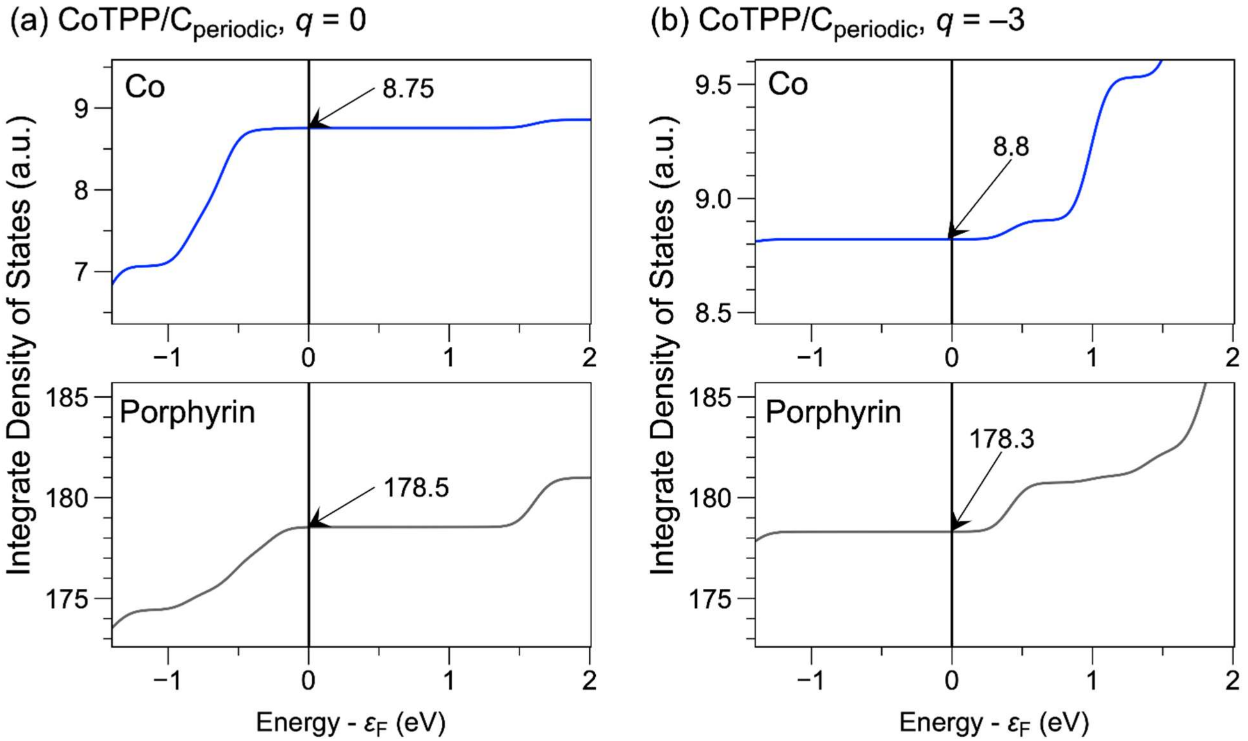

Figure S7. Integrated DOS of Co and porphyrin related electronic states for CoTPP/C$_{periodic}$ model with (a) no added charge and (b) with three added electrons. Labels indicate the value of the integrated DOS at the Fermi energy (ε_F). The value of the integrated DOS at ε_F corresponds to the number of electrons occupying Co or porphyrin related states. Porphyrin related states include contributions from carbon, nitrogen, and hydrogen related states.

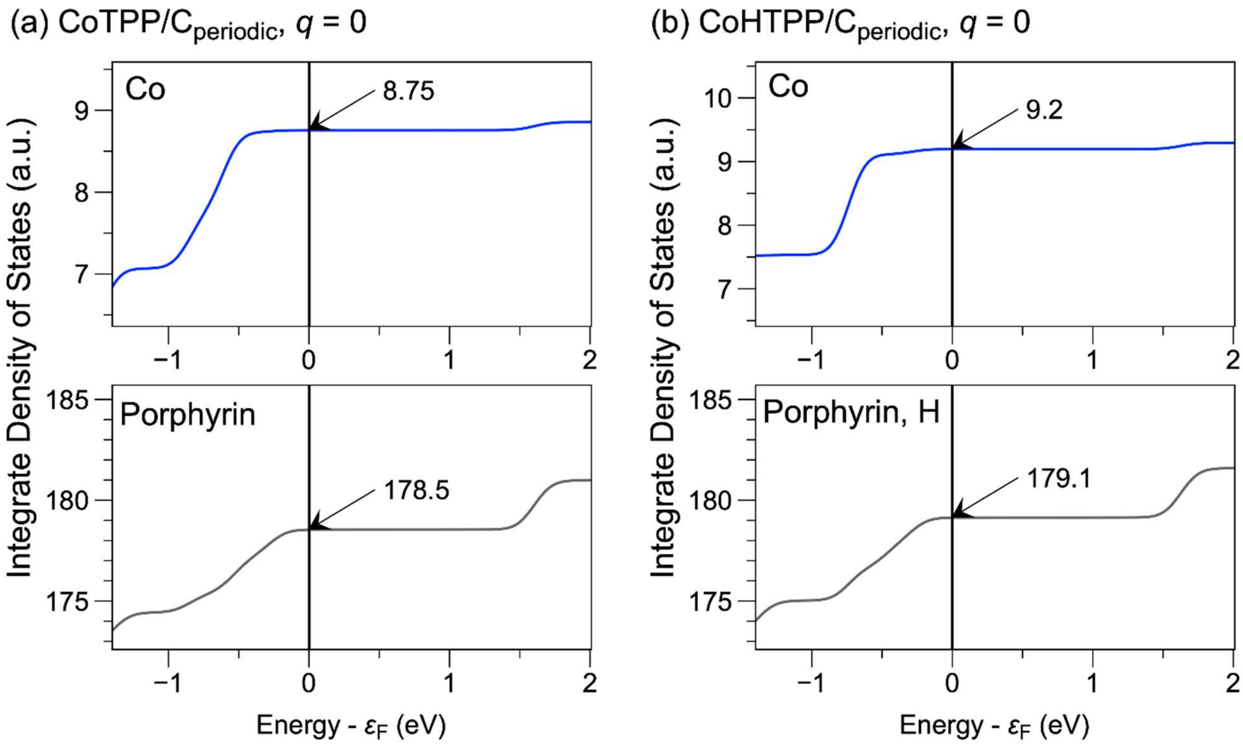

Figure S8. Integrated DOS of Co and porphyrin related electronic states for (a) CoTPP/$C_{periodic}$ model and (b) CoHTPP/$C_{periodic}$ model. Labels indicate the value of the integrated DOS at the Fermi energy (ε_F). The electronic states related to the added hydrogen are summed into the porphyrin related electronic states. The change in occupancy amounts to 1.05 electrons.

Density of States for Periodic Models with Axial Ligation

Ligation of the Co(II)TPP to an oxidic defect in the periodic models (CoTPP-O-$C_{periodic}$) results in changes to the Co(II)TPP electronic structure, which is evident in the PDOS as a cobalt related state directly below the Fermi energy. This behavior is analogous to that in the graphitic flake models, where the HOMO is mixed between the graphitic surface and CoTPP. This change in cobalt electronic structure is due to overlap between the cobalt (blue in Figure S9a) and oxygen (red in Figure S9a) electronic states and causes spin density to be distributed to the oxygen from the cobalt, as discussed further below. The Co related states (blue) shift with the oxygen related states (red) in the PDOS upon addition of electronic charge (Figure S9b) due to strong electronic coupling between them. The species formed through PCET (CoHTPP-O-$C_{periodic}$, Figure S9c) shows a distribution of d-states reminiscent of a cobalt hydride, although there is still some coupling to the oxygen. This coupling is evident by the slight overlap between the oxygen related

states and the Co related states in Figure S9c. We identify oxidation states based on the system net spin and the projection of the wavefunction onto the cobalt d-states.

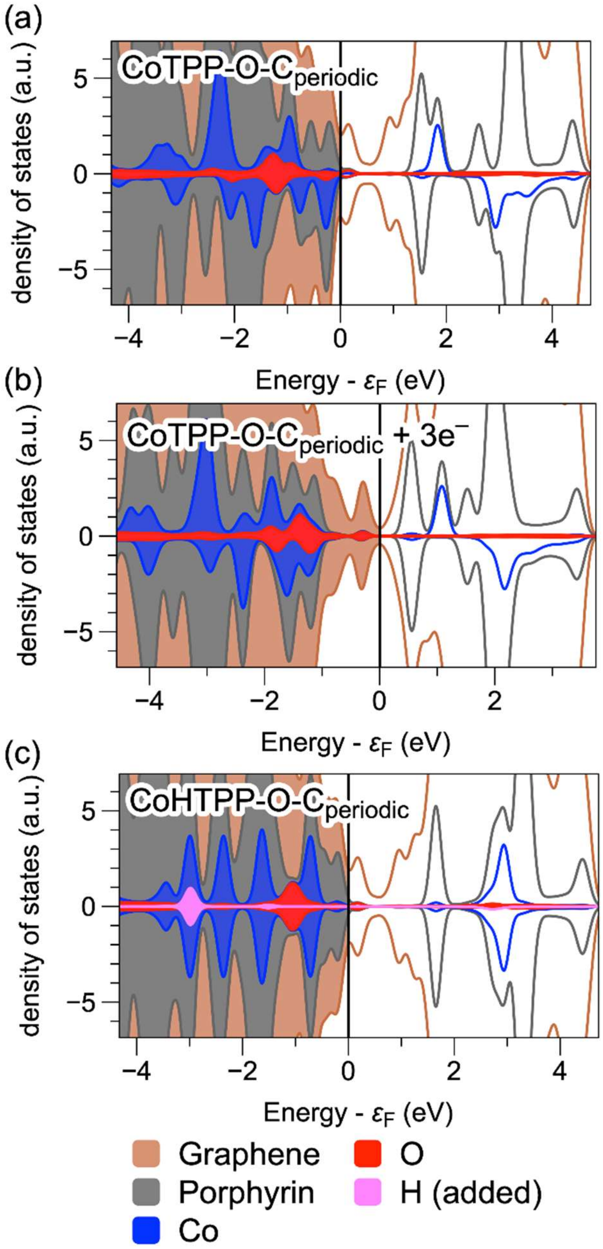

Figure S9. PDOS for periodic models with CoTPP axially ligated to an oxidic defect on the graphene basal plane. (a) CoTPP-O-$C_{periodic}$ with no added charge; (b) CoTPP-O-$C_{periodic}$ with three electrons added; (c) CoHTPP-O-$C_{periodic}$, produced after adding one proton and one electron to CoTPP-O-$C_{periodic}$.

CoTPP Spin Density in Periodic Models

The spin densities in the periodic models are consistent with those of the cluster-based models, indicating that the oxidation states are likely similar. In the π-stacked model, the spin density exhibits strong d_{z^2} character, indicating a singly occupied d_{z^2} orbital, which is consistent with the cluster-based models. For the axially ligated model, the spin density is less clear and is partially delocalized onto the oxygen and carbon, as in the cluster-based models. Both spin densities are shown in Figure S10.

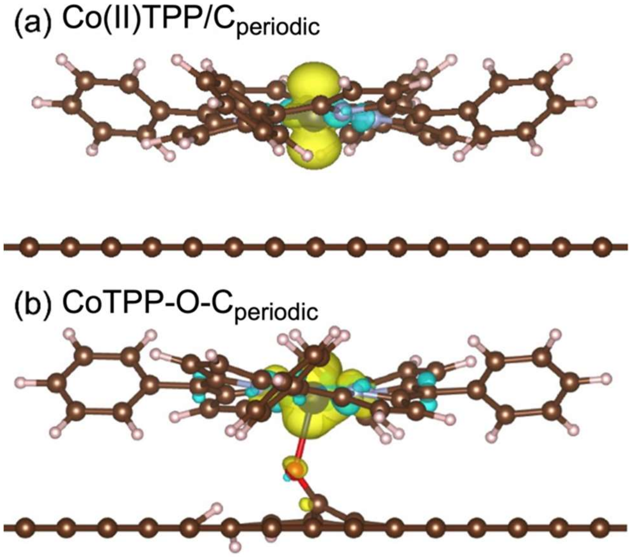

Figure S10. Spin densities for the periodic graphitic models (a) Co(II)TPP/$C_{periodic}$ and (b) CoTPP-O-$C_{periodic}$. Isosurfaces plotted with a value of 0.002 $Å^{-3}$.

Cobalt Oxidation State and d-States

For the cluster models, we determined the Mulliken spin population of the cobalt atom in all systems studied and performed a localized orbital bonding analysis (LOBA)[17, 18] to determine the cobalt oxidation state. These values are given in Table S2 for the various models, including the molecular cobalt porphyrin in solution.

Table S2. Mulliken Spin Populations and LOBA Determined Oxidation State of the Cobalt in Cluster Models of Adsorbed CoTPP.

System	Mulliken Spin Population	Co Oxidation State
Molecular Co(II)TPP	1.124	2
Molecular Co(I)TPP	0.000	1
Molecular Co(III)HTPP	0.000	3
Co(II)TPP/$C_{96}H_{26}$	1.122	2
Co(II)TPP/$[C_{96}H_{26}]^{-}$	1.122	2
Co(III)HTPP/$[C_{96}H_{26}]^{+}$	0.000	3
Co(III)HTPP/$C_{96}H_{26}$	0.000	3
Co(III)TPP-O_{edge}-$C_{80}H_{21}$	0.000	3
[Co(II)TPP-O_{edge}-$C_{80}H_{21}]^{-}$	0.964	2
Co(III)HTPP-O_{edge}-$C_{80}H_{21}$	0.019	3
[Co(III)HTPP-O_{edge}-$C_{80}H_{21}]^{-}$	0.000	3
CoTPP-O_{basal}-$C_{80}H_{22}$	0.483	3
[CoTPP-O_{basal}-$C_{80}H_{22}]^{-}$	0.000	3
Co(III)HTPP-O_{basal}-$C_{80}H_{22}$	0.000	3

For the periodic models, the projection of the wavefunction onto the cobalt d-states was analyzed in an effort to identify the cobalt oxidation state. In the periodic models, the alpha and beta spin states of the cobalt d-states are different energy (i.e., spin polarized). The identification of the oxidation states is challenging for the CoTPP/$C_{periodic}$ and CoTPP-O-$C_{periodic}$ systems, as the d-states can be highly degenerate and show non-integer occupation (Figure S11). We assume oxidation states that are consistent with the results from the analogous cluster-based calculations when the occupation of the d-states and net magnetization of the system are not definitive.

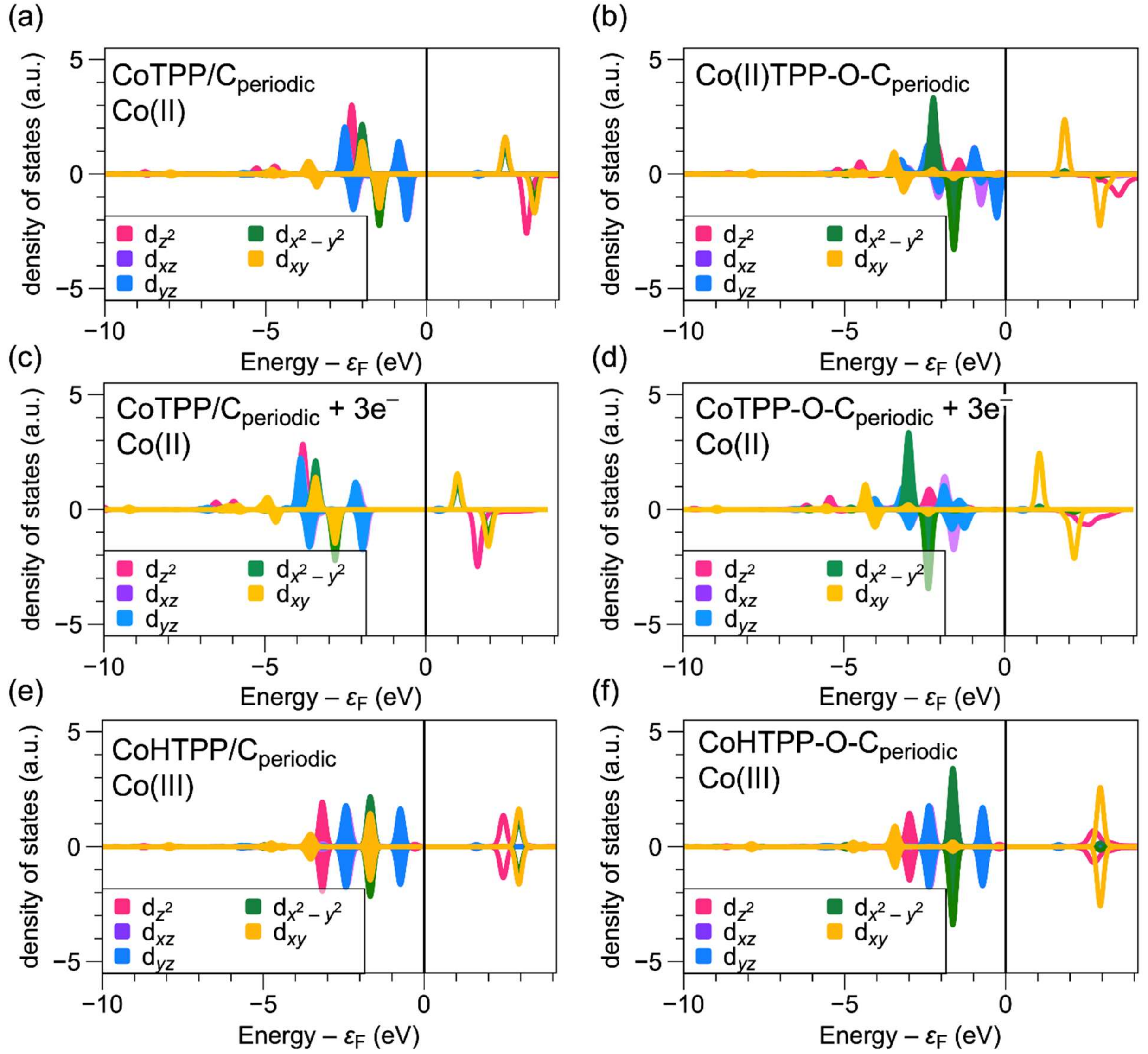

Figure S11. Projection of the wavefunction onto the cobalt d-states for π-stacked and axially ligated periodic models of CoTPP adsorbed on graphene. (a) d-states for CoTPP/$C_{periodic}$ showing three unoccupied states and a singly occupied d_{z^2}. (b) d-states for CoTPP-O-$C_{periodic}$ have ambiguous occupation. (c) d-states for CoTPP/$C_{periodic}$ + 3e⁻ show no change in cobalt oxidation or occupation of the d_{z^2}. (d) d-states for CoTPP-O-$C_{periodic}$ + 3e⁻ suggest the CoTPP oxidation state is unaltered. (e) d-states for CoHTPP/$C_{periodic}$, which is assigned as Co(III)-H. (f) d-states for CoHTPP-O-$C_{periodic}$ are fairly consistent with CoHTPP/$C_{periodic}$ and thus are also assigned as Co(III)-H.

Decay of Electrostatic Potential between CoTPP and Graphene

Electrostatic coupling between the CoTPP and the graphitic surface can be illustrated by analyzing the change in the interfacial electrostatic potential that occurs when electrons are added to the electrode surface. As presented in the main text, charging of the electrode changes the electrostatic potential at the adsorbed CoTPP due to electrostatic coupling between the molecule and the surface (Figure S12a). Upon increasing the separation between CoTPP and graphene, this electrostatic coupling decreases, as shown in Figure S12b. The decrease in electrostatic coupling is evident as the graphitic surface experiences the same amount of polarization, yet the polarization on the CoTPP is significantly less. At these separations, long-range electrostatic interactions are still non-zero but are much smaller. With the increased separation, both implicit solvent and the counter charge can be placed between the CoTPP and graphene (Figure S12b). Moving the counter charge to the other side of the CoTPP produces a qualitatively similar but slightly altered picture (Figure S12e). Moreover, the features observed in the calculations including a strip of counter charge are qualitatively similar but slightly altered when a homogeneous background charge is employed instead (Figure S12c-d). The calculations with only a homogeneous background charge are more susceptible to a spurious net dipole to the overall unit cell and thus are considered to be less reliable for these types of systems.

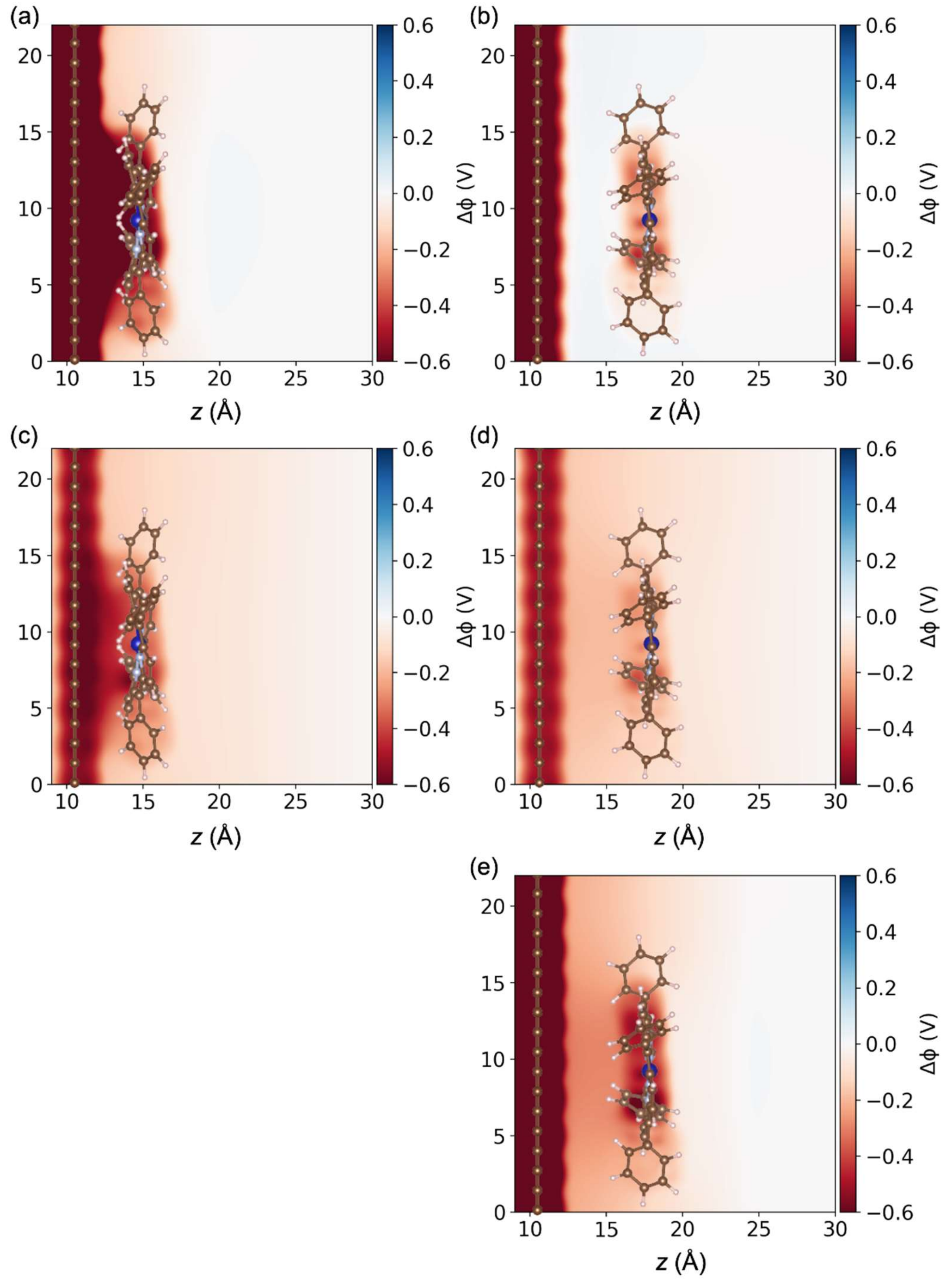

Figure S12. Changes in the electrostatic potential caused by the addition of three electrons to periodic models for (a) CoTPP adsorbed on graphene, with a separation between CoTPP and graphene of ~3.4 Å; (b) CoTPP is separated from graphene by ~6.5 Å and the counter charge is placed at z = 14 Å; (c) CoTPP is adsorbed on graphene and a homogeneous background charge neutralizes the unit cell; (d) CoTPP is separated from graphene by ~6.5 Å and a homogeneous background charge neutralizes the unit cell; (e) CoTPP is separated from graphene by ~6.5 Å and the counter charge is placed at z = 25 Å. Red indicates a more negative electrostatic potential in the system with three electrons than in the neutral system.

References

(1) Frisch, M. J.; Trucks, G. W.; Schlegel, H. B.; Scuseria, G. E.; Robb, M. A.; Cheeseman, J. R.; Scalmani, G.; Barone, V.; Petersson, G. A.; Nakatsuji, H., et al. *Gaussian 16 Rev. C.01*, Wallingford, CT, 2016.

(2) Ditchfield, R.; Hehre, W. J.; Pople, J. A. Self-Consistent Molecular-Orbital Methods. IX. An Extended Gaussian-Type Basis for Molecular-Orbital Studies of Organic Molecules. *J. Chem. Phys.* **1971,** *54*, 724-728.

(3) Francl, M. M.; Pietro, W. J.; Hehre, W. J.; Binkley, J. S.; Gordon, M. S.; DeFrees, D. J.; Pople, J. A. Self-consistent molecular orbital methods. XXIII. A polarization-type basis set for second-row elements. *J. Chem. Phys.* **1982,** *77*, 3654-3665.

(4) Hariharan, P. C.; Pople, J. A. The influence of polarization functions on molecular orbital hydrogenation energies. *Theor. Chim. Acta.* **1973,** *28*, 213-222.

(5) Hariharan, P. C.; Pople, J. A. Accuracy of AH n equilibrium geometries by single determinant molecular orbital theory. *Mol. Phys.* **1974,** *27*, 209-214.

(6) Hehre, W. J.; Ditchfield, R.; Pople, J. A. Self—Consistent Molecular Orbital Methods. XII. Further Extensions of Gaussian—Type Basis Sets for Use in Molecular Orbital Studies of Organic Molecules. *J. Chem. Phys.* **1972,** *56*, 2257-2261.

(7) Rassolov, V. A.; Pople, J. A.; Ratner, M. A.; Windus, T. L. 6-31G* basis set for atoms K through Zn. *J. Chem. Phys.* **1998,** *109*, 1223-1229.

(8) Rassolov, V. A.; Ratner, M. A.; Pople, J. A.; Redfern, P. C.; Curtiss, L. A. 6-31G* basis set for third-row atoms. *J. Comput. Chem.* **2001,** *22*, 976-984.

(9) Hay, P. J.; Wadt, W. R. Ab initio effective core potentials for molecular calculations. Potentials for K to Au including the outermost core orbitals. *J. Chem. Phys.* **1985,** *82*, 299-310.

(10) Wadt, W. R.; Hay, P. J. Ab initio effective core potentials for molecular calculations. Potentials for main group elements Na to Bi. *J. Chem. Phys.* **1985,** *82*, 284-298.

(11) Barone, V.; Cossi, M. Quantum Calculation of Molecular Energies and Energy Gradients in Solution by a Conductor Solvent Model. *J. Phys. Chem. A* **1998,** *102*, 1995-2001.

(12) Cossi, M.; Rega, N.; Scalmani, G.; Barone, V. Energies, structures, and electronic properties of molecules in solution with the C-PCM solvation model. *J. Comput. Chem.* **2003,** *24*, 669-681.

(13) Dal Corso, A. Pseudopotentials periodic table: From H to Pu. *Cmput. Mater. Sci.* **2014,** *95*, 337-350.

(14) Giannozzi, P.; Andreussi, O.; Brumme, T.; Bunau, O.; Buongiorno Nardelli, M.; Calandra, M.; Car, R.; Cavazzoni, C.; Ceresoli, D.; Cococcioni, M., et al. Advanced capabilities for materials modelling with Quantum ESPRESSO. *J. Phys. Condens. Matter.* **2017,** *29*, 465901.

(15) Giannozzi, P.; Baroni, S.; Bonini, N.; Calandra, M.; Car, R.; Cavazzoni, C.; Ceresoli, D.; Chiarotti, G. L.; Cococcioni, M.; Dabo, I., et al. QUANTUM ESPRESSO: a modular and open-source software project for quantum simulations of materials. *J. Phys. Condens. Matter.* **2009,** *21*, 395502.

(16) Andreussi, O.; Dabo, I.; Marzari, N. Revised self-consistent continuum solvation in electronic-structure calculations. *J. Chem. Phys.* **2012,** *136*, 064102.

(17) Thom, A. J. W.; Sundstrom, E. J.; Head-Gordon, M. LOBA: a localized orbital bonding analysis to calculate oxidation states, with application to a model water oxidation catalyst. *Phys. Chem. Chem. Phys.* **2009,** *11*, 11297-11304.

(18) LU Tian, C. F.-W. Calculation of Molecular Orbital Composition. *Acta. Chim. Sin.* **2011,** *69*, 2393-2406.
